# Molecular Profiling of Circulating Tumour Cells Identifies Notch1 as a Principal Regulator in Advanced Non-Small Cell Lung Cancer

**DOI:** 10.1038/srep37820

**Published:** 2016-11-30

**Authors:** Javier Mariscal, Marta Alonso-Nocelo, Laura Muinelo-Romay, Jorge Barbazan, Maria Vieito, Alicia Abalo, Antonio Gomez-Tato, Casares de Cal Maria de los Angeles, Tomas Garcia-Caballero, Carmela Rodriguez, Elena Brozos, Francisco Baron, Rafael Lopez-Lopez, Miguel Abal

**Affiliations:** 1 Translational Medical Oncology, Health Research Institute of Santiago (IDIS), University Hospital of Santiago (SERGAS), Trav. Choupana s/n 15706 Santiago de Compostela, Spain.; 2 Liquid Biopsy Analysis Unit, Health Research Institute of Santiago (IDIS), University Hospital of Santiago (SERGAS), Trav. Choupana s/n 15706 Santiago de Compostela, Spain; 3 School of Mathematics, University of Santiago de Compostela (Campus Vida), C/Lope Gomez de Marzoa s/n 15782 Santiago de Compostela, Spain; 4 Department of Morphological Sciences, School of Medicine, University of Santiago de Compostela, Spain

## Abstract

Knowledge on the molecular mechanisms underlying metastasis colonization in Non-Small Cell Lung Cancer (NSCLC) remains incomplete. A complete overview integrating driver mutations, primary tumour heterogeneity and overt metastasis lacks the dynamic contribution of disseminating metastatic cells due to the inaccessibility to the molecular profiling of Circulating Tumour Cells (CTCs). By combining immunoisolation and whole genome amplification, we performed a global gene expression analysis of EpCAM positive CTCs from advanced NSCLC patients. We identified an EpCAM+ CTC-specific expression profile in NSCLC patients mostly associated with cellular movement, cell adhesion and cell-to-cell signalling mediated by PI3K/AKT, ERK1/2 and NF-kB pathways. *NOTCH1* emerged as a driver connecting active signalling pathways, with a reduced number of related candidate genes (*NOTCH1, PTP4A3, LGALS3* and *ITGB3*) being further validated by RT-qPCR on an independent cohort of NSCLC patients. In addition, these markers demonstrated high prognostic value for Progression-Free Survival (PFS). In conclusion, molecular characterization of EpCAM+ CTCs from advanced NSCLC patients provided with highly specific biomarkers with potential applicability as a “liquid biopsy” for monitoring of NSCLC patients and confirmed *NOTCH1* as a potential therapeutic target to block lung cancer dissemination.

Cancer is a major public health concern in developed countries with lung cancer as the leading cause responsible for 19.4% of cancer deaths worldwide^1^. Of those, Non-Small Cell Lung Cancer (NSCLC) accounts for 85% of all cases and encompasses high genetic and histological heterogeneity^2^. Clinically, treatment alternatives attains a 54% 5-year survival when disease is localized but only 4% when disease is diagnosed once metastasis has occurred^3^. In this scenario, and despite of the increase in survival during the last decade due to the incorporation of new drugs and better treatment selection^4^, NSCLC remains an incurable disease in the metastatic setting. Since more than 58% of patients present locally advanced or metastatic disease at the moment of diagnosis^3^, strategies to understand and target the metastatic process constitute a priority in NSCLC.

Circulating Tumour Cells (CTCs) represent a principal component of the step-wise process of metastasis in which tumour cells escape from the primary tumour, invade the vascular and lymphatic systems, and incorporate into the circulation. Some of these cells are able to survive in the absence of physical substrate until, eventually, reattach at a distant organ and colonize it. Only recently, expanded CTCs *ex vivo* from early stage lung cancer have been observed to carry the same genetic alterations as primary tumors^5^; and when transplanted CTCs have been able to generate new tumour masses in mouse model while maintaining the genetic and biological characteristics of the origin tumor^6^. Moreover, CTCs enumeration has proven to correlate with poor prognosis in several cancers including NSCLC^7^,^8^.

To this regard, CTC evaluation allows readily “liquid biopsy” meanwhile access to surgical and/or biopsy specimens is often insufficient and fails to reflect tumour dynamics, heterogeneity or even drug sensitivity. Therefore, CTCs represent a powerful tool for disease management in agreement with the importance of developing non-invasive biomarkers to monitor tumour dynamics at real-time^9^.

In this work we present a methodology for the recovery of EpCAM positive CTCs from advanced NSCLC patients, followed by whole genome amplification and gene expression array analysis to characterize this metastatic population. Although EpCAM expression is frequently lost during cancer progression in NSCLC, EpCAM expression in CTCs correlates with poorer outcome in previous studies^8^, while EpCAM negative CTCs failed to prove prognostic value in lung cancer^10^. As from these data we were able to describe a panel of genes specifically expressed in CTCs from advanced NSCLC patients, and to improve our understanding on the biology of the subpopulation of CTCs in NSCLC. Moreover, we characterized NOTCH1 as main signalling pathway that illustrate their fate, and identified potential biomarkers with clinical significance.

## Results

### Immunoisolation and molecular profiling of CTC from advanced NSCLC patients

**Figure 1:**
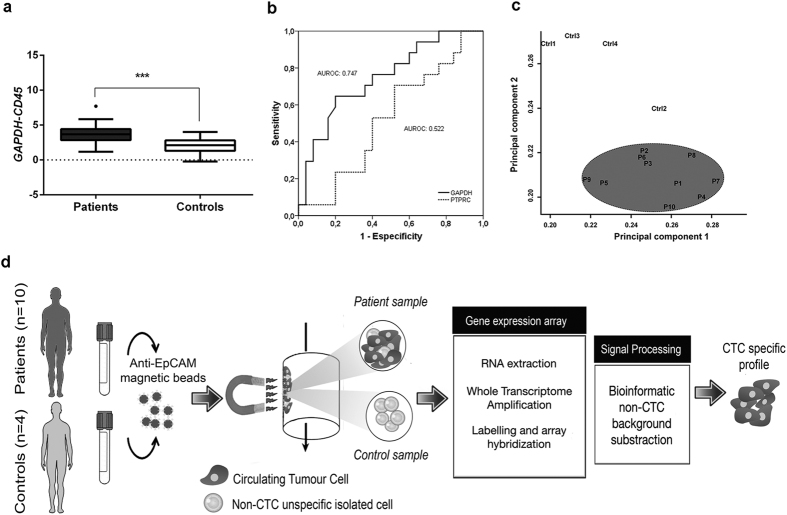
CTC detection and gene expression profiling. **(a)** *GADPH* expression in CTC immunoisolated from advanced NSCLC patient (n = 42) and control (n = 16) groups. **(b)** AUROC curve for *GAPDH* and *CD45* detection for RT-qPCR. Unspecific-isolated lymphocytes do not predict early progression (<4 months). **(c)** Principal component dot plot for differential distribution of NSCLC patients and healthy donors based on gene expression microarray. NSCLC patients are grouped constituting a particular population. **(d)** Schematic workflow for CTC gene expression profiling.

The presence of CTCs in advanced NSCLC was assessed by combining EpCAM-based immunoisolation and RT-qPCR analysis of *GAPDH* in a series of 42 advanced NSCLC patients (Supplementary Table S1) and 16 healthy donors. *GAPDH* has been stated as a reference marker of global cellularity when normalized to the recovery of *CD45* as a marker of the non-specific isolated lymphocytes^11^, herein demonstrating the presence of an additional population in advanced NSCLC patients (p < 0.001; Fig. 1a). Also consistently, *CD45* expression showed no significant differences when evaluated for both groups revealing same unspecific recovery in patients and controls (data not shown) and no power was observed for early progression in the cohort of patients (AUROC: 0.522) while *GAPDH* presented an AUROC 0.747 (Fig. 1b). Additionally, when *CD45* gene expression was assessed in the remaining cellular fraction upon CTC immunoisolation no significant differences were found between patients and controls, revealing that CTC specific gene expression is not associated with differential expression in the non-CTC population^11^.

Molecular profiling of CTCs in advanced NSCLC was approached by subtracting the background of unspecific immunoisolation from healthy donors (n = 4) to the global gene expression profile of EpCAM-based immunoisolated CTCs from patients (n = 10; Supplementary Table S2), as described by Barbazan *et al*.^11^. Experimental workflow for CTC isolation and enrichment, RNA extraction, whole transcriptome amplification and array hybridization is depicted in Fig. 1d. Total RNA from CTCs was amplified, complementary DNA was hybridized onto Agilent gene expression microarray, and raw data was pre-processed as described in Material and Methods, resulting in 2,392 spots (7.01%) that accomplished the quality criteria. Average signal was 60,889 units being 76,597 and 16,979 units the average signal for patient and control groups respectively. Upon normalization 1,810 probes with expression greater than 2^11^ in at least 8 patients and 2 controls were considered for any further analysis. Assuming Delta parameter to 2.46 for median False Discovery Rate (FDR) = 0 rendered 853 differentiated probes; being considered only the 305 probes with log2 ratio greater than 1.5 (Supplementary Table S3). In addition, 8 probes uniquely detected in patients’ samples, with a raw signal meeting the quality criteria, were included into subsequent analysis (Supplementary Table S4). Principal Component Analysis (PCA) revealed effective discrimination between patients and control samples, NSCLC patients being defined by their pathologic status (Fig. 1c).

### Biology of CTC isolated from advanced NSCLC patients

**Figure 2:**
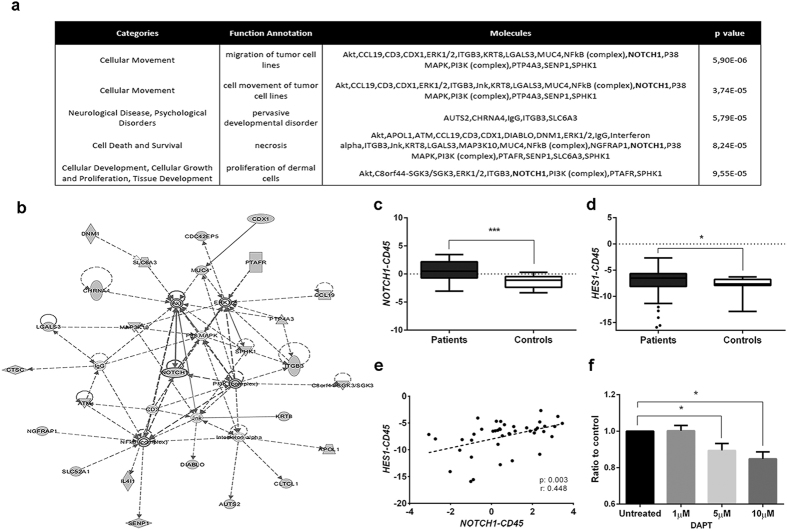
CTC expression profile analysis and *NOTCH1* validation. **(a)** Cellular functions and biological networks by IPA for CTC from advanced NSCLC patients. **(b)** Interaction network for cell movement, cell death and survival, cell development and cell growth and proliferation according to CTC expression profile. *NOTCH1* is identified as a main actor orchestrating the network. **(c)** Validation of specific *NOTCH1* gene expression in CTC from advanced NSCLC patients (n = 42) as compared to controls (n = 16). **(d)** *HES1* gene expression is significantly increased in NSCLC patients compared to controls. **(e)** *NOTCH1* correlates with his effector protein HES1 in NSCLC CTCs. **(f)** Clonogenic assay for A549 adenocarcinoma cells treated with the γ-secretase inhibitor DAPT (n = 6).

Bioinformatic analysis with Ingenuity Pathway Analysis (IPA) software was performed to interpret the biology of NSCLC CTCs. Consistently with an actively disseminating subpopulation of tumour cells, the main molecular and cellular functions represented by the set of CTC-specific genes were related to cellular movement, cell death and survival and cellular development, cellular growth and proliferation, mainly orchestrated by PI3K/AKT, ERK1/2 and NF-kB pathways. GeneCodis3 ontology analysis also highlighted cell adhesion, developmental functions, extracellular matrix organization and an active transduction and transport activity (data not shown). Interestingly, *NOTCH1* was identified as an important actor orchestrating this functional network (Fig. 2a and b). The biological and functional relevance of *NOTCH1* within this metastatic population was further confirmed by RT-qPCR (Fig. 2c) in the series of 42 NSCLC patients (Supplementary Table S1). In addition, we assessed the expression of *HES1*, a downstream target gene of *NOTCH1*, whose expression was found to be significantly increased in patients (p = 0.041; Fig. 2d), in correlation with that of *NOTCH1* (Fig. 2e). Moreover, we further assessed the functional relevance of Notch1 in the subpopulation of CTC in NSCLC by studying the impact of Notch1 pathway inhibition on clonogenic assay, as an indirect measure of the metastatic potential of CTC. As shown in Fig. 2f, the γ-secretase inhibitor DAPT significantly reduced the ability of A549 cells to grow in the absence of substrate at different concentrations *in vitro* (p = 0.031).

We finally screened the expression levels of *LGALS3* (Galectin-3), *ITGB3* (Integrin beta-3) and *PTP4A3* (Protein tyrosine phosphatase type IVA 3), associated with the identified *NOTCH1* network, in CTC immunoisolated from NSCLC patients. RT-qPCR evaluation showed a significant increased expression of the selected three candidate genes in the series of CTCs immunoisolated from patients with advanced NSCLC when compared to healthy donors (p < 0.05; Supplementary Figure S1). All these results confirmed the relevance of *NOTCH1* in NSCLC dissemination and validated the bioinformatics analysis and biological information of the gene expression profiling of EpCAM+ CTCs immunoisolated from advanced NSCLC patients.

### Prognostic significance of Notch1 network in CTCs from NSCLC

CTC gene expression profiling from advanced NSCLC patients also provided with diagnostic and prognostic markers with potential clinical impact in patient management. Univariate Cox regression analysis showed prognostic utility for *NOTCH1* (p = 0.034), *PTP4A3* (p = 0.003), *LGALS3* (p = 0.044) and *ITGB3* (p = 0.046) to predict Progression-Free Survival, and that for *ITGB3* (p = 0.006) for Overall Survival. The poor prognosis groups were defined within the restrictive 33% of patients with the highest RT-qPCR signal for each biomarker (Supplementary Table S5, upper panel). Moreover, *GAPDH* proved clinical value to predict PFS (p < 0.001) and OS (p < 0.001) as expected for an indirect evaluator of the CTC burden (Supplementary Table S5, upper panel). Additionally, the presence of bone metastasis was identified as the only clinical parameter with prognostic significance in series of 42 advanced NSCLC patients (Supplementary Table S5, lower panel).

**Figure 3:**
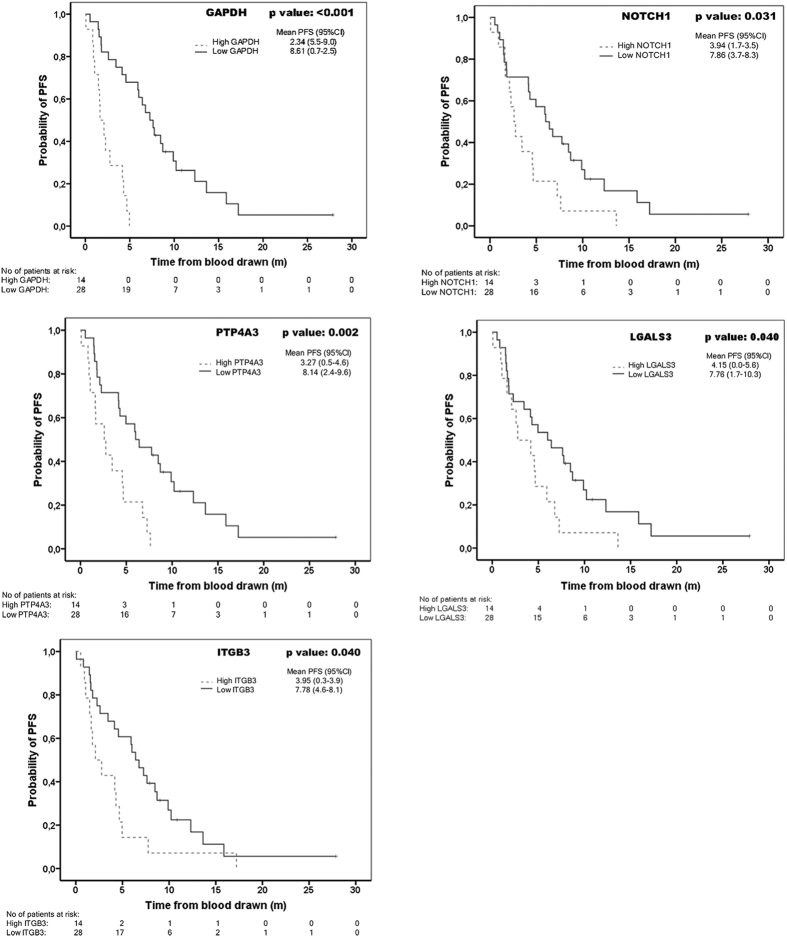
Kaplan-Meier curves of validated biomarkers for Progression-Free Survival (PFS). Statistical significance determined by log rank test (p < 0.05).

**Figure 4:**
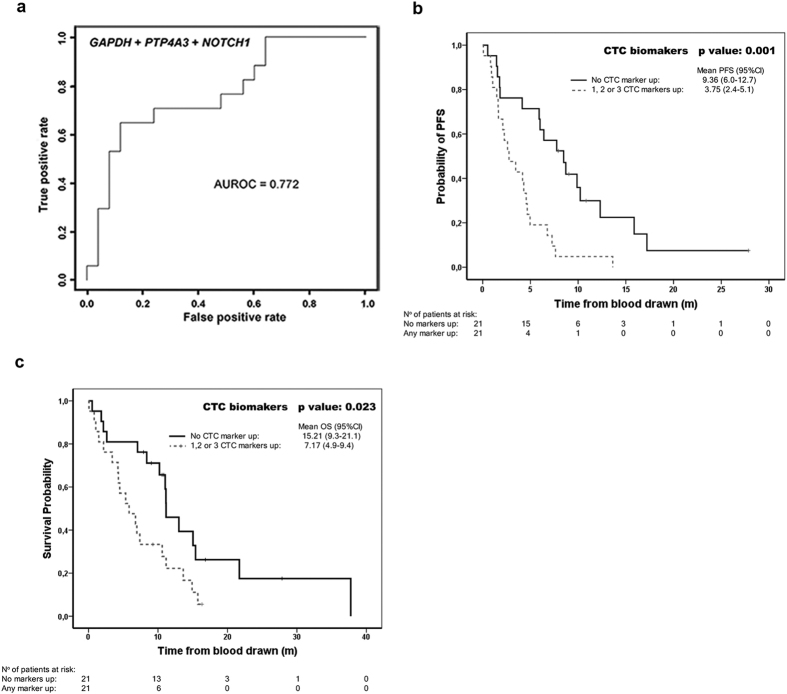
Prognosis and diagnosis value for the accumulation of biomarkers and logistic model. **(a)** Linear regression model for *GAPDH* + *NOTCH1* + *PTP4A3* biomarkers. (**b** and **c**) Kaplan-Meier analysis grouping patients according to the marrkers included in the regression model for **(b)** PFS and **(c)** OS.

Kaplan-Meier analysis confirmed the prognostic value of *NOTCH1, PTP4A3, LGALS3, ITGB3*, and *GAPDH* biomarkers for PFS (Fig. 3), and that for *GAPDH* and *ITGB3* for OS (Supplementary Figure S2). We further generated a logistic model with the panel of CTC-biomarkers in order to improve their diagnostic and prognostic value. For this, linear regression with a 67% good prognosis versus 33% poor prognosis cut-off for each biomarker as previously defined, demonstrated the best performance with an AUROC value of 0.772 for the combination of *GAPDH, NOTCH1* and *PTP4A3* in early progression (Fig. 4a). Accordingly, the prognostic value of the panel of CTC-biomarkers improved for PFS and OS when defined as a cumulative variable for the markers included in the logistic model (Fig. 4b,c).

Finally, multivariate analysis including the panel of five CTC-biomarkers in combination with the presence of bone metastasis resulted in *GAPDH, NOTCH1* and *PTP4A3* as independent predictors of PFS (Table 1, upper panel); *GAPDH* and *ITGB3* showed predictive value for OS (Table 1, upper panel). When referred to the cumulative variable of the logistic model CTC-biomarker panel, predictive value was achieved for PFS and OS, as well as bone metastasis (Table 1, lower panel).

## Discussion

Research on circulating tumour cells (CTCs) has rapidly increased in the last years in response to the clinical need of new alternative tools for the management of patients candidate for personalized therapeutic strategies^12^. Traditionally, efforts have been devoted to understand the mechanisms underlying cancer progression mainly focused on primary and metastatic carcinomas; but little is known about the “fluid phase” of cancer as the relevant actor of tumour dissemination and lethality^13^. This is particularly relevant in NSCLC, where most cases result inoperable and biopsies are rarely obtainable. The analysis and characterization of CTCs in NSCLC patients represent a particularly attractive strategy for real-time patient monitoring to support treatment decisions^14^,^15^.

CTCs exist in extreme rarity in blood; therefore their analysis requires highly sensitive methods. In the last decade a number of technologies, not only restricted to immunoisolation, have become available^16^. Most of the commonly used strategies for CTC detection showed promising results in NSCLC, although the amount of data available is limited compared to other tumour types^17^,^18^. For instance, studies with the FDA-approved CellSearch system demonstrated utility for CTC detection with high levels of CTCs at baseline correlating with poor survival rates^19^,^20^; although heterogeneity in EpCAM expression could limit its applicability in NSCLC patients^21^. To this regard, we must also consider that EpCAM is frequently overexpressed in NSCLC, suggesting an implication of EpCAM in carcinogenesis^22^. In addition, EpCAM positivity was confirmed in our series of samples showing strong immunostaining in well-differentiated tumour areas while positivity was moderate in poorly differentiated tumour areas (Supplementary Figure S3). In this work, we focus on EpCAM-positive CTC from advanced NSCLC patients, and we approached their global gene expression profiling to better understand their biology and to identify and validate CTC-biomarkers with clinical utility in NSCLC.

Previous studies on CTC gene expression in NSCLC approached PCR-based technologies analysing subsets of genes from CTC enriched samples^23^,^24^. We combined CTC immunoisolation, whole genome amplification and gene-expression profiling. The specific NSCLC-derived CTCs expression profile is mainly associated with cellular movement, cell adhesion and differentiation, and cell-to-cell signalling linked to PI3K/AKT, ERK1/2 and NF-kB pathways, previously associated with lung cancer and metastasis^25^,^26^; and *NOTCH1* playing an important role on the whole functional network. Of note, among genes related to cell adhesion we could find tenascin R (*TNR)* or mucin 4 (*MUC4)*, previously associated with lung cancer aggressiveness and dissemination^27^,^28^.

Notch signalling is emerging as a valuable molecular pathway for targeting therapies in NSCLC. Clinical studies indicate that 30% of NSCLC cases have increased Notch1 signaling^29^ and this activity seems to be critical for driving cancer cell migration and metastasis due to repression of cell adhesion molecules, resulting in loss of cell-to-cell adhesion, protection against hypoxic conditions and promotion of a stem-like signature^30^. Recent studies have demonstrated that Notch signalling plays an important role in lung cancer initiation via crosstalk with transcription factors enhancing epithelial-to-mesenchymal transition (EMT)^31^. Further, dysregulation of Notch signalling as a common feature of NSCLC correlating with poor prognosis has been associated with TGF-β and EMT^32^. Related to this, Notch1 related EMT promotion has been associated with an increased EpCAM expression^33^. Of note, Notch signalling inhibition resulting in a non-significant decreased EpCAM expression (Supplementary Figure S3), did not altered the efficiency of EpCAM-based immunoisolation of CTC (Supplementary Figure S3). These results indicate that EpCAM-based immunoisolation is effective to purify CTC from NSCLC patients, the excess of antibody coupled beads are in excess being able to balance the variations in EpCAM expression due to Notch1-related EMT or the presence of poorly differentiated tumour cells (Supplementary Figure S3). Similarly, no differences in Notch1 were found between CTC-EpCAM positive immunoisolated from adenocarcinoma or squamous cell carcinoma origins (data not shown), although specific studies should be performed to address the impact of histology or mutational profiles associated with smoking^34^. Also accordingly, *HES1* downstream target of *NOTCH1*, strongly correlated in the CTCs immunoisolated from advanced NSCLC patients, further suggesting the functional involvement of Notch pathway and the potential therapeutic value of Notch pathway-directed drugs such as γ-secretase inhibitors or ADAM inhibitors in advanced NSCLC^35^,^36^.

In addition to *NOTCH1*, with demonstrated prognostic value in NSCLC^37^, validated genes involved in the identified CTC-network have also been described during NSCLC progression. The expression of the growth/adhesion-regulatory lectin galectin-3 in stage II NSCLC is indicative of a poor prognosis^38^,^39^, playing a role in cell motility, invasion, and metastasis^40^. Similarly, PTP4A3 expression in NSCLC has been described upregulated in correlation with advanced clinical stage and distant metastasis linked to microvascular and lymphatic vessel formation^41^. Finally, ITGB3 has been recently described as mediator of NSCLC promotion in the context of K-ras addiction^42^, and whose inhibition by miR-98^43^ or miR let-7c^44^ prevented proliferation, migration and invasion in NSCLC.

The promising results obtained with the panel of CTC-biomarkers in NSCLC both for detection of metastatic disease and classification into favourable and unfavourable outcome guarantee further analysis in a large multicenter study including therapy monitoring.

In conclusion, we demonstrated that the combination of CTC immunoisolation and gene expression analyses represents an adequate strategy to: a) identify highly specific and sensitive CTC biomarkers with independent diagnosis and prognosis value and potential applicability in the management of advanced NSCLC patients; b) reveals *NOTCH1* as a conducting factor in the biology of EpCAM positive CTCs and a potential therapeutic target to impair metastasis dissemination in NSCLC.

## Material and Methods

### Patient selection

Participants included in the study were diagnosed with advanced stage (unresectable stage III or IV) NSCLC on the basis of the International Association for the Study of Lung Cancer (IASLC)/ American Thoracic Society (ATS)/European Respiratory Society (ERS) classification criteria. Blood samples were collected before the initiation of chemotherapy, in accordance to the guidelines and protocols approved by the Institutional Ethical Committee (Galician Clinical Research Ethics Committee, SERGAS; code 2008/277), and signed informed consent was obtained from all participants. Candidates who had received chemotherapy previously to the study were not included.

CTCs evaluation cohort included 42 NSCLC patients with distant metastasis diagnosed in 36 cases (Supplementary Table S1). Microarray gene-expression analysis was conducted in an independent set of patients and healthy age-matched donors. Distant metastasis was present in 8 out of 10 patients at the moment of recruitment (Supplementary Table S2).

### CTC immunoisolation, profiling and Significance Analysis Microarray

CTC isolation was performed from 7.5 ml of peripheral blood from advanced NSCLC patients or healthy donors using the EpCAM-based CELLection^TM^ Epithelial Enrich Dynabeads® kit (Invitrogen, Dynal; Oslo, Norway) according to manufacturers’ instructions. Isolated CTCs were resuspended in RNA later® (Ambion; Foster City, CA, USA) and preserved at −80°C until use.

Total RNA extraction, Complete Whole Transcriptome Amplification (WTA2, Sigma Aldrich) and gene expression array was performed as described by Barbazan *et al*.^11^. Briefly, total RNA was extracted with the QIAmp viral RNA mini kit (Qiagen, Valencia, CA, USA) specifically designed for very low cellularity samples. Subsequent pure RNA was then subjected to Complete Whole Transcriptome Amplification PCR for 20 cycles using the maximum amount of RNA; Cy3 labelling and hybridization onto Agilent 4 × 44 k gene expression arrays. Upon hybridization, signal was captured and submitted to Significance Analysis for Microarray to identify differentially expressed genes (Supplementary Material and Methods S1).

### Ingenuity Pathway Analysis (IPA) and Gene Ontology analysis

Gene set characterizing CTC population was analysed with IPA software for representative biological pathways and functions in our data set. 204 probes were identified as annotated genes (66.6%). Fisher’s exact test was applied to calculate p value of biological related functions.

Additionally, CTC specific gene signature was submitted to gene ontology analysis using GeneCodis3 open access software^45^. Singular enrichment Gene Ontology tool was used to obtain a broad overview of the main biological processes involved in the CTC profile; of which 193 probes belonged to annotated genes (61.1%). Hypergeometrical test (p < 0.05) and FDR correction were used as stated in the software.

### Preamplification and quantitative Real-Time PCR validation

CTCs were isolated from an independent set of patients and total RNA was extracted with the QIAmp viral RNA mini kit (Qiagen; Valencia, CA, USA) as previously stated. Complementary DNA (cDNA) was obtained using SuperScript III reverse transcriptase (Invitrogen; Carlsbad, CA, USA) adding 11 μl of total RNA per reaction. Because of very low cellularity of the samples, cDNA was subjected to 14x pre-amplification cycles (TaqMan® PreAmp Master Mix Kit, Applied Biosystems; Foster City, CA, USA) to increase sensibility and stability. Finally, pre-amplified 1:10 diluted cDNA was subjected to TaqMan RT-qPCR for candidate genes associated with Notch1 network by IPA (Supplementary Table S4). *GAPDH*, a reference gene previously used for CTC quantification in peripheral blood and indirect evidence of cellularity^11^,^46^, and *HES1* as a downstream gene of *NOTCH1* (Supplementary Material and Methods S1) were also included. Mean threshold cycle (depicted as 40-Ct) for every candidate gene was normalized to *CD45*, a lymphocyte specific marker that allows the quantification of non-specific isolation and that have been previously used to discriminate the population of CTCs from the total of nucleated cells isolated in blood^11^,^47^,^48^. Samples were run in duplicate and all plates included negative controls.

### Cell culture

The human NSCLC cell line A549 was obtained from the American Type Culture Collection. A549 cells were maintained in Dulbecco’s Modified Eagle Medium (DMEM) with 10% FBS (Invitrogen). Cells were kept at 37 °C under a humidified atmosphere with 5% CO_2_. Mycoplasma test was conducted routinely.

### Soft agar assay and Notch1 inhibition

Soft agar assays were conducted on 96-well plates with a base layer of 0.6% low melting point agarose and an upper layer of 0.3% low melting point agarose containing 5 × 10^3^ cells per well. Upper and lower layer were constituted in 2% FBS DMEM.

Inhibition of Notch1 was approached by the addition of 100 μl of the γ-secretase inhibitor, DAPT (CAS 208255-80-5, Santa Cruz Biotechnology, Dallas, TX, USA; IC_50_: 115 nM), or its vehicle (DMSO) at different concentrations in FBS-free DMEM. Proliferation was measured five days later by AlamarBlue® Cell Viability Reagent (DAL1025, ThermoFisher). Significance was evaluated by Wilcoxon signed rank test (p < 0.05).

### Statistical validation and clinical database contrast

Validation analysis was performed using two-tailed Mann-Whitney U-test. Clinical evaluation of validated markers was approached following the REMARK recommendations^49^. Progression-Free Survival (PFS) and Overall Survival (OS) were analysed using Kaplan-Meier analysis and differences were examined by log rank test. Univariate and multivariate analyses were performed using Cox regression statistics. Bivariate correlation analysis was routinely carried out according Pearson statistic. p < 0.05 values were considered statistically significant.

## Additional Information

**How to cite this article**: Mariscal, J. *et al*. Molecular Profiling of Circulating Tumour Cells Identifies Notch1 as a Principal Regulator in Advanced Non-Small Cell Lung Cancer. *Sci. Rep.* **6**, 37820; doi: 10.1038/srep37820 (2016).

**Publisher's note:** Springer Nature remains neutral with regard to jurisdictional claims in published maps and institutional affiliations.

## Supplementary Material

Supplementary Information

## Figures and Tables

**Table 1:** Multivariate Cox regression analysis for CTC markers and clinicopathological parameters individually and in combination.

Multivariate Cox analysis	Progression-Free Survival		Overall Survival	
	HR (95% CI)	p value	HR (95% CI)	p value
*GAPDH*				
* GAPDH* (poor vs. good prognosis)	5.67 (2.1–15.6)	**0.001**	10.29 (3.4–30.7)	**<0.001**
* *Bone metastasis (Y/N)	1.16 (0.5–2.7)	0.727	0.74 (0.3–1.8)	0.503
*NOTCH1*				
* NOTCH1* (poor vs. good prognosis)	2.20 (1.1–4.4)	**0.027**	1.22 (0.6–2.5)	0.582
* *Bone metastasis (Y/N)	2.48 (1.2–5.0)	**0.011**	1.86 (0.9–3.7)	0.087
*PTP4A3*				
* PTP4A3* (poor vs. good prognosis)	3.17 (1.5–6.7)	**0.002**	1.57 (0.8–3.2)	0.222
* *Bone metastasis (Y/N)	2.45 (1.2–4.9)	**0.012**	1.92 (0.9–3.9)	0.073
*LGALS3*				
* LGALS3* (poor vs. good prognosis)	1.93 (0.9–3.9)	0.067	1.59 (0.8–3.2)	0.204
* *Bone metastasis (Y/N)	2.29 (1.1–4.6)	**0.021**	1.81 (0.9–3.7)	0.101
*ITGB3*				
* ITGB3* (poor vs. good prognosis)	1.72 (0.8–3.5)	0.134	2.72 (1.3–5.6)	**0.007**
* *Bone metastasis (Y/N)	2.12 (1.0–4.3)	**0.040**	1.59 (0.8–3.3)	0.211
CTC-biomarker panel				
* *1–3 biomarkers (poor vs. good prognosis)	3.06 (1.5–6.3)	**0.002**	2.33 (1.1–4.8)	**0.022**
* *Bone metastasis (Y/N)	2.22 (1.1–4.5)	**0.028**	1.91 (0.9–3.9)	0.077

*HR: hazard ratio; CI: confidence interval.*
