# Supplementary material for: Molecular Profiling of Circulating Tumour Cells Identifies Notch1 as a Principal Regulator in Advanced Non-Small Cell Lung Cancer
Source: Sci Rep. 2016 Nov 30;6:37820. doi: 10.1038/srep37820 (PMC5129014; doi:10.1038/srep37820)
Supplement: Supplementary Information [file srep37820-s1.pdf]

MOLECULAR PROFILING OF CIRCULATING TUMOUR CELLS IDENTIFIES NOTCH1 AS A PRINCIPAL REGULATOR IN ADVANCED NON-SMALL CELL LUNG CANCER

Javier Mariscal[1], Marta Alonso-Nocelo[1], Laura Muinelo-Romay[1,2], Jorge Barbazan[1], Maria Vieito[1], Alicia Abalo[1,2], Antonio Gomez-Tato[3], Maria de los Angeles Casares de Cal[3], Tomas Garcia-Caballero[4], Carmela Rodriguez[1], Elena Brozos[1], Francisco Baron[1], Rafael Lopez-Lopez[1,2] and Miguel Abal[1,*]

[1]*Translational Medical Oncology, Health Research Institute of Santiago (IDIS). University Hospital of Santiago (SERGAS). Trav. Choupana s/n 15706 Santiago de Compostela, Spain.*

[2]*Liquid Biopsy Analysis Unit, Health Research Institute of Santiago (IDIS). University Hospital of Santiago (SERGAS). Trav. Choupana s/n 15706 Santiago de Compostela, Spain.*

[3]*School of Mathematics, University of Santiago de Compostela (Campus Vida). C/Lope Gomez de Marzoa s/n 15782 Santiago de Compostela, Spain.*

[4]*Department of Morphological Sciences, School of Medicine, University of Santiago de Compostela, Spain.*

*** Corresponding author:** Miguel Abal, Translational Medical Oncology (IDIS/SERGAS); Trav. Choupana s/n 15706; Santiago de Compostela (Spain); phone: +34 981955073; Email: miguel.abal.posada@sergas.es

SUPPLEMENTARY MATERIAL AND METHODS

Significance Analysis for Microarrays

Upon microarray hybridization, signal was captured and processed using an Agilent scanner (G2565B, Agilent Technologies). The scanner images were segmented by the Agilent Feature Extraction Software (v9.5) with the protocol GE1-v5_95. Extended dynamic range implemented in the Agilent software was applied to avoid saturation in the highest intensity range. The processed signal (gProcessed-Signal) value was chosen for the statistical analysis instead of the signal with subtracted background (gBGSubSignal) since it produces lower average coefficient of variation (CV) in Spike-In and gene replicates[1,2].

The following features and/or genes which did not conform to the established quality criteria were filtered: (a) non-uniform pixel distributed outliers and population replicate outliers according to the default Agilent feature extraction criteria; (b) spots not differentiated from background signal (as estimated for each spot); (c) spots in the range of negative controls.

Normalization among all microarray data was performed by the quantile method implemented in the limma package of the Bioconductor statistical software[3]. Only signals above 2^{11} after normalization were considered to reliably interpret the gene expression information. In addition, probes with no signal in more than one patient were not included in the analysis. Finally, two class unpaired SAM algorithm implemented in the R package samr[4] was used to identify differentially expressed genes.

1. Klebanov, L. & Yakovlev, A. How high is the level of technical noise in microarray data? *Biol. Direct* **2,** 9 (2007).
2. Zahurak, M. *et al.* Pre-processing Agilent microarray data. *BMC Bioinformatics* **8,** 142 (2007).
3. Ritchie, M. E. *et al.* limma powers differential expression analyses for RNA-sequencing and

microarray studies. *Nucleic Acids Res.* **43,** e47 (2015).

4. Tibshirani, A. R., Chu, G., Narasimhan, B. & Li, J. Package samr. SAM: Significance Analysis of Microarrays: R package version 2.0. (2011). at <http://cran.r-project.org/package=samr>

EpCAM-based A549 isolation assay and EpCAM expression

2.5x10^5 A549 lung cancer cells were plated and serum-deprived for 16h. Cells were treated or not with the γ-secretase inhibitor DAPT 20µM (CAS 208255-80-5, Santa Cruz Biotechnology) for 8 or 24 hours, and 10^4 cells were harvested and added to 7.5 ml of buffer 2 (Ca^{2+} and Mg^{2+} free PBS, BSA 0.1%) as referred for CTC immunoisolation. Isolation protocol was conducted according to manufacturer' instructions (CELLection™ Epithelial Enrich, Invitrogen). Cells were incubated with 100 µM of EpCAM-functionalized beads for 30' (4°). Finally, immunoisolated cells were lysed, total RNA extracted (Qiamp Viral RNA mini kit, Qiagen) and cDNA synthetized according to provider's protocol (Superscript III, Invitrogen). Subsequent, cDNA was 1:8 diluted and GADPH expression determined by RT-qPCR as housekeeping for cellularity.

EpCAM expression was analysed by immunohistochemistry as previously described [5]. Alternatively, EpCAM expression upon Notch signalling inhibition assessed by HES1 and HEY1 expression, was performed after similar DAPT treatment, total RNA extraction (High Pure RNA isolation kit, Roche), cDNA synthesis and RT-qPCR evaluation. All measurements included duplicates and negative controls. Fold change was established after normalization to GAPDH expression and significance determined by Wilcoxon signed rank test ($p<0.05$).

5. Alonso-Alconada, L. *et al.* Molecular profiling of circulating tumor cells links plasticity to the metastatic process in endometrial cancer. *Molecular Cancer* **13**, 223 (2014)

TaqMan RT-qPCR probes

GAPDH	Human	Hs99999905_m1	NM_002046.4	122
CD45	Human	Hs00894734_m1	NM_002829.3	70
NOTCH1	Human	Hs01062014_m1	NM_017617.3	80
HES1	Human	Hs00172878_m1	NM_005515.1	78
PTP4A3	Human	Hs02341135_m1	NM_007079.3	112
			NM_116000.1	112
LGALS3		Hs00173587_m1	NM_001177388.1	64
			NM_002306.3	64
ITGB3	Human	Hs01001469_m1	NM_000212.2	59

SUPPLEMENTARY TABLE S1

Clinicopathological parameters of the cohort of NSCLC patients

**Sex**	Male	33	78.6
	Female	9	21.4
**Smoking habits**	Smoker (current & former)	34	91.9
	Non smoker	3	8.1
**Histology**	Adenocarcinoma	32	76.2
	Squamous cell carcinoma	10	23.8
**Tumour differentiation**	Well	2	4.8
	Moderate	10	23.8
	Undifferentiated	9	21.4
	Unknown	21	50.0
**Tumour stage**	IIIA	3	7.1
	IIIB	3	7.1
	IV	36	85.7
**Tumour size (T)**	T0	2	4.9
	T1	5	12.2
	T2	9	22.0
	T3	7	17.1
	T4	18	43.9
**Node affectation (N)**	N0	5	11.9
	N1	1	2.4
	N2	16	38.1
	N3	20	47.6
**No. of focus of metastasis**	None	6	14.3
	Low (<2)	12	28.6
	High (≥2)	24	57.1
**Metastasis location**	Lung	15	35.7
	Bone	14	33.3
	Liver	9	21.4
	Supra-adrenal	10	23.8
	Other	11	26.2
**Chemotherapy**	Platinum doublet	38	92.7
	GEM-VNB	1	2.4
	ERLOTINIB	2	4.9

% as cumulative percentage

SUPPLEMENTARY TABLE S2

Clinicopathological parameters of the patients included in gene expression microarrays

**Patient 1**	M	74	Smoker	Squamous cell	IIIB	T4	N3	M0	-	Platinum doublet
**Patient 2**	M	70	Smoker	Squamous cell	IIIB	T4	N3	M0	-	Platinum doublet
**Patient 3**	M	62	Smoker	Adenocarc.	IV	T3	N3	M1	Brain, lymph node	-
**Patient 4**	F	61	Unknown	Adenocarc.	IV	T2	N2	M1	Brain, lung	Platinum doublet
**Patient 5**	M	67	Smoker	Squamous cell	IV	T4	N2	M1	Pancreas, supra-adrenal	GEM-VNB
**Patient 6**	M	53	Smoker	Adenocarc.	IV	T3	N3	M1	Lymph node, bone, pleural	Platinum doublet
**Patient 7**	M	53	Smoker	Adenocarc.	IV	T4	N3	M1	Lung, liver, lymph node, bone	Platinum doublet
**Patient 8**	M	73	Smoker	Adenocarc.	IV	T3	N2	M1	Liver, bone	Platinum doublet
**Patient 9**	M	60	Smoker	Squamous cell	IV	T4	N1	M1	Bone, supra-adrenal, pleural	Platinum doublet
**Patient 10**	M	68	Smoker	Squamous cell	IV	T3	N3	M1	Liver	Platinum doublet

G: gender; T: tumor size; N: node affectation; M: presence of metastasis

SUPPLEMENTARY TABLE S3

Genes statistically more detected in NSCLC patients from Aqilent gene expression microarrays

** ratio calculated as the ratio between the mean signal between patients and controls*

ALG1	A_32_P169131	ref|Homo sapiens asparagine-linked glycosylation 1, beta-1,4-mannosyltransferase homolog (S. cerevisiae) (ALG1), mRNA [NM_019109]	4,411	5,95E-04
LOC100132147	A_24_P631848	gb|Homo sapiens cDNA clone IMAGE:4816083, partial cds. [BC036435]	4,260	6,21E-04
PRB4	A_33_P3216150	ref|Homo sapiens proline-rich protein BstNI subfamily 4 (PRB4), mRNA [NM_002723]	4,171	5,96E-04
KIAA1875	A_33_P3209541	ref|Homo sapiens KIAA1875 (KIAA1875), non-coding RNA [NR_024207]	4,089	7,33E-04
LOC643669	A_24_P822692	gb|PREDICTED: Homo sapiens similar to mCG2980, transcript variant 1 (LOC643669), mRNA [XM_933621]	4,008	6,62E-04
SLC4A1	A_33_P3400217	ref|Homo sapiens solute carrier family 4, anion exchanger, member 1 (erythrocyte membrane protein band 3, Diego blood group) (SLC4A1), mRNA [NM_000342]	3,989	8,06E-04
LOC644992	A_33_P3329352	ref|PREDICTED: Homo sapiens hypothetical LOC644992 (LOC644992), mRNA [XM_001717733]	3,980	6,06E-04
MFSD10	A_23_P41246	ref|Homo sapiens major facilitator superfamily domain containing 10 (MFSD10), transcript variant 1, mRNA [NM_001120]	3,884	8,89E-04
A_33_P3271187	A_33_P3271187	Unknown	3,873	6,03E-04
CLEC16A	A_33_P3422888	gb|Homo sapiens C-type lectin domain family 16, member A, mRNA (cDNA clone MGC:111457 IMAGE:4419248), complete cds. [BC112897]	3,737	5,91E-04
ENST00000398726	A_33_P3230037	ens|Putative uncharacterized protein ENSP00000381711 Fragment [Source:UniProtKB/TrEMBL;Acc:A8MYH6] [ENST00000398726]	3,526	5,62E-04
psiTPTE22	A_33_P3213149	gb|Homo sapiens mRNA for Hypothetical protein FLJ37713 variant, clone: ah02419. [AK226145]	3,511	7,10E-04
LOC440795	A_33_P3218120	gb|PREDICTED: Homo sapiens similar to hCG1646661 (LOC440795), mRNA [XM_001719381]	3,439	8,28E-04

LCE1D	A_23_P375524	ref|Homo sapiens late cornified envelope 1D (LCE1D), mRNA [NM_178352]	3,332	7,58E-04
A_33_P3321682	A_33_P3321682	Unknown	3,309	1,25E-03
OLFML2A	A_33_P3248137	ref|Homo sapiens olfactomedin-like 2A (OLFML2A), mRNA [NM_182487]	3,309	5,74E-04
LOC400968	A_33_P3235132	gb|Homo sapiens cDNA FLJ45884 fis, clone OCBBF3021166. [AK127783]	3,299	5,72E-04
LOC100132716	A_33_P3352048	gb|Homo sapiens cDNA FLJ46328 fis, clone TESTI4044291. [AK128833]	3,279	7,27E-04
A_33_P3347522	A_33_P3347522	Unknown	3,204	7,07E-04
AX747590	A_33_P3245858	gb|Sequence 1115 from Patent EP1308459. [AX747590]	3,176	6,51E-04
KRTAP10-10	A_33_P3381851	ref|Homo sapiens keratin associated protein 10-10 (KRTAP10-10), mRNA [NM_181688]	3,174	6,47E-04
FLJ40606	A_33_P3303031	gb|Homo sapiens hypothetical protein LOC643549, mRNA (cDNA clone IMAGE:40147028). [BC133006]	3,122	5,70E-04
BC137015	A_24_P15640	gb|Homo sapiens chromosome 19 open reading frame 31, mRNA (cDNA clone MGC:168631 IMAGE:9021008), complete cds. [BC137015]	3,105	8,20E-04
LOC100292717	A_33_P3298552	ref|PREDICTED: Homo sapiens similar to arg tyrosine kinase (LOC100292717), mRNA [XM_002345084]	3,098	6,75E-04
OGFR	A_23_P28707	ref|Homo sapiens opioid growth factor receptor (OGFR), mRNA [NM_007346]	3,097	6,16E-04
LOC100131551	A_33_P3228732	ref|Homo sapiens hypothetical LOC100131551 (LOC100131551), non-coding RNA [NR_024480]	3,093	7,98E-04
ENST00000344692	A_33_P3285715	ens|zinc finger protein 41 homolog (mouse) [Source:HGNC Symbol;Acc:26786] [ENST00000344692]	3,076	6,79E-04
MON1B	A_33_P3308626	ref|Homo sapiens MON1 homolog B (yeast) (MON1B), mRNA [NM_014940]	3,072	6,44E-04
IL4I1	A_33_P3405424	ref|Homo sapiens interleukin 4 induced 1 (IL4I1), transcript variant 2, mRNA [NM_172374]	3,061	6,46E-04
CASKIN1	A_33_P3349552	ref|Homo sapiens CASK interacting protein 1 (CASKIN1), mRNA [NM_020764]	3,039	5,68E-04
CSNK1E	A_33_P3265185	ens|casein kinase 1, epsilon [Source:HGNC Symbol;Acc:2453] [ENST00000403904]	3,038	5,55E-04

GPSM1	A_33_P3331687	ref|Homo sapiens G-protein signaling modulator 1 (AGS3-like, C. elegans) (GPSM1), transcript variant 1, mRNA [NM_001145638]	3,036	1,74E-03
LOC644242	A_33_P3596355	gb|602301760F1 NIH_MGC_87 Homo sapiens cDNA clone IMAGE:4403366 5', mRNA sequence [BG033631]	2,998	6,18E-04
PPP1R14A	A_33_P3335682	ref|Homo sapiens protein phosphatase 1, regulatory (inhibitor) subunit 14A (PPP1R14A), mRNA [NM_033256]	2,997	9,53E-04
C1orf229	A_24_P831309	ref|Homo sapiens chromosome 1 open reading frame 229 (C1orf229), mRNA [NM_207401]	2,993	2,49E-03
TOR2A	A_33_P3275835	ref|Homo sapiens torsin family 2, member A (TOR2A), transcript variant 3, mRNA [NM_001134430]	2,983	5,68E-04
RPH3A	A_33_P3395384	ref|Homo sapiens rabphilin 3A homolog (mouse) (RPH3A), transcript variant 1, mRNA [NM_001143854]	2,983	5,58E-04
LHX3	A_33_P3223678	ref|Homo sapiens LIM homeobox 3 (LHX3), transcript variant 2, mRNA [NM_014564]	2,971	5,55E-04
AK090448	A_33_P3292864	gb|Homo sapiens mRNA for FLJ00369 protein. [AK090448]	2,968	6,97E-04
A_33_P3393370	A_33_P3393370	Unknown	2,965	6,27E-04
A_33_P3218741	A_33_P3218741	Unknown	2,944	5,78E-04
LOC100130456	A_33_P3231572	gb|Homo sapiens cDNA FLJ37693 fis, clone BRHIP2014954. [AK095012]	2,918	5,86E-04
LOC100132815	A_32_P85042	ens|cDNA FLJ32177 fis, clone PLACE6001294HCG2000535 ; [Source:UniProtKB/TrEMBL;Acc:Q96ML8] [ENST00000397094]	2,916	5,60E-04
PRB1	A_33_P3258593	ref|Homo sapiens proline-rich protein BstNI subfamily 1 (PRB1), transcript variant 1, mRNA [NM_005039]	2,903	8,42E-04
KRT7	A_33_P3350748	ref|Homo sapiens keratin 7 (KRT7), mRNA [NM_005556]	2,901	9,75E-04
LRRC46	A_23_P152949	ref|Homo sapiens leucine rich repeat containing 46 (LRRC46), mRNA [NM_033413]	2,892	5,61E-04
SLC14A2	A_23_P27353	ref|Homo sapiens solute carrier family 14 (urea transporter), member 2 (SLC14A2), mRNA [NM_007163]	2,881	6,10E-04
FLJ36000	A_33_P3377763	ref|Homo sapiens hypothetical FLJ36000 (FLJ36000), non-coding RNA [NR_027084]	2,881	6,23E-04
SPHK1	A_33_P3222069	ref|Homo sapiens sphingosine kinase 1 (SPHK1), transcript variant 2, mRNA [NM_182965]	2,872	1,40E-03

KIRREL2	A_33_P3420852	ref|Homo sapiens kin of IRRE like 2 (Drosophila) (KIRREL2), transcript variant 3, mRNA [NM_199180]	2,869	7,71E-04
MCCD1	A_23_P133904	ref|Homo sapiens mitochondrial coiled-coil domain 1 (MCCD1), nuclear gene encoding mitochondrial protein, mRNA [NM_001011700]	2,860	5,57E-04
MR1	A_23_P431939	ref|Homo sapiens major histocompatibility complex, class I-related (MR1), mRNA [NM_001531]	2,859	7,46E-04
SPRR2D	A_33_P3322388	ref|Homo sapiens small proline-rich protein 2D (SPRR2D), mRNA [NM_006945]	2,844	5,90E-04
ENST00000324659	A_33_P3415859	ens|NLR family, CARD domain containing 3 [Source:HGNC Symbol;Acc:29889] [ENST00000324659]	2,837	7,58E-04
LOC389834	A_33_P3364293	ref|Homo sapiens ankyrin repeat domain 57 pseudogene (LOC389834), non-coding RNA [NR_027420]	2,818	6,32E-04
DNM1	A_33_P3329419	ref|Homo sapiens dynamin 1 (DNM1), transcript variant 1, mRNA [NM_004408]	2,807	9,52E-04
LTBP4	A_33_P3348091	ref|Homo sapiens latent transforming growth factor beta binding protein 4 (LTBP4), transcript variant 1, mRNA [NM_001042544]	2,759	7,55E-04
KCNH3	A_33_P3253672	ref|Homo sapiens potassium voltage-gated channel, subfamily H (eag-related), member 3 (KCNH3), mRNA [NM_012284]	2,754	7,62E-04
THC2647746	A_33_P3403851	thc|O52K2_HUMAN (Q8NGK3) Olfactory receptor 52K2, partial (29%) [THC2647746]	2,749	6,37E-04
**NOTCH1**	**A_33_P3370424**	**ref|Homo sapiens Notch homolog 1, translocation-associated (Drosophila) (NOTCH1), mRNA [NM_017617]**	**2,744**	**6,84E-04**
MCC	A_33_P3288246	ref|Homo sapiens mutated in colorectal cancers (MCC), transcript variant 1, mRNA [NM_001085377]	2,740	6,35E-04
A_33_P3399911	A_33_P3399911	Unknown	2,724	6,91E-04
CATSPERG	A_33_P3620488	ref|Homo sapiens cation channel, sperm-associated, gamma (CATSPERG), mRNA [NM_021185]	2,720	6,20E-04
GNPTG	A_23_P14886	ref|Homo sapiens N-acetylglucosamine-1-phosphate transferase, gamma subunit (GNPTG), mRNA [NM_032520]	2,720	6,01E-04
DLK1	A_33_P3217559	ref|Homo sapiens delta-like 1 homolog (Drosophila) (DLK1), mRNA [NM_003836]	2,716	1,10E-03
CRYBA2	A_33_P3314231	ref|Homo sapiens crystallin, beta A2 (CRYBA2), transcript variant 1, mRNA [NM_005209]	2,713	6,00E-04
A_33_P3365963	A_33_P3365963	Unknown	2,696	7,49E-04

BCL2L15	A_33_P3408177	ref|Homo sapiens BCL2-like 15 (BCL2L15), mRNA [NM_001010922]	2,695	7,06E-04
NHLH2	A_33_P3231252	ref|Homo sapiens nescient helix loop helix 2 (NHLH2), transcript variant 1, mRNA [NM_005599]	2,694	2,02E-03
A_33_P3370515	A_33_P3370515	Unknown	2,682	1,12E-03
ENST00000398625	A_33_P3420914	ens|Putative uncharacterized protein ENSP00000381622Putative uncharacterized protein ENSP00000381624; [Source:UniProtKB/TrEMBL;Acc:A8MWS4] [ENST00000398625]	2,677	6,98E-04
NKX1-2	A_24_P256155	ref|Homo sapiens NK1 homeobox 2 (NKX1-2), mRNA [NM_001146340]	2,671	7,41E-04
PLEKHF1	A_23_P79134	ref|Homo sapiens pleckstrin homology domain containing, family F (with FYVE domain) member 1 (PLEKHF1), mRNA [NM_024310]	2,670	6,94E-04
FLJ44653	A_33_P3408244	gb|Homo sapiens FLJ44653 protein, mRNA (cDNA clone MGC:168579 IMAGE:9020956), complete cds. [BC136964]	2,663	5,77E-04
SFXN5	A_33_P3292896	ref|Homo sapiens sideroflexin 5 (SFXN5), mRNA [NM_144579]	2,655	6,27E-04
FLJ44477	A_33_P3229452	gb|Homo sapiens cDNA FLJ44477 fis, clone UTERU2031703. [AK126441]	2,646	9,32E-04
ATXN2L	A_33_P3369956	ref|Homo sapiens ataxin 2-like (ATXN2L), transcript variant E, mRNA [NM_148416]	2,644	7,73E-04
A_33_P3301097	A_33_P3301097	Unknown	2,641	9,20E-04
GOLGA6L9	A_33_P3243878	ref|Homo sapiens golgin A6 family-like 9 (GOLGA6L9), mRNA [NM_198181]	2,630	6,15E-04
MAP3K10	A_24_P284523	ref|Homo sapiens mitogen-activated protein kinase kinase kinase 10 (MAP3K10), mRNA [NM_002446]	2,623	6,92E-04
CYTH2	A_23_P119377	ref|Homo sapiens cytohesin 2 (CYTH2), transcript variant 2, mRNA [NM_004228]	2,621	9,01E-04
CLDN19	A_33_P3299739	ref|Homo sapiens claudin 19 (CLDN19), transcript variant 2, mRNA [NM_001123395]	2,612	6,12E-04
CDC42EP5	A_33_P3342235	ref|Homo sapiens CDC42 effector protein (Rho GTPase binding) 5 (CDC42EP5), mRNA [NM_145057]	2,609	9,93E-04
DCLK2	A_33_P3237734	ref|Homo sapiens doublecortin-like kinase 2 (DCLK2), transcript variant 1, mRNA [NM_001040260]	2,608	5,79E-04
SLC6A3	A_33_P3303772	ref|Homo sapiens solute carrier family 6 (neurotransmitter transporter, dopamine), member 3 (SLC6A3), mRNA [NM_001044]	2,606	9,15E-04

BC012881	A_33_P3298750	gb|Homo sapiens cDNA clone IMAGE:3463076. [BC012881]	2,605	6,40E-04
TNR	A_23_P45864	ref|Homo sapiens tenascin R (restrictin, janusin) (TNR), mRNA [NM_003285]	2,601	7,52E-04
GPR172B	A_23_P15692	ref|Homo sapiens G protein-coupled receptor 172B (GPR172B), transcript variant 2, mRNA [NM_017986]	2,598	6,49E-04
EPHX3	A_23_P119593	ref|Homo sapiens epoxide hydrolase 3 (EPHX3), transcript variant 1, mRNA [NM_024794]	2,591	6,28E-04
PLEKHG5	A_33_P3272539	ref|Homo sapiens pleckstrin homology domain containing, family G (with RhoGef domain) member 5 (PLEKHG5), transcript variant 2, mRNA [NM_198681]	2,584	5,73E-04
ENST00000398387	A_33_P3385561	ens|cDNA FLJ27422 fis, clone WMC08087Putative uncharacterized protein ENSP00000381423; [Source:UniProtKB/TrEMBL;Acc:Q6ZNN6] [ENST00000398387]	2,583	8,60E-04
BSX	A_33_P3311210	ref|Homo sapiens brain-specific homeobox (BSX), mRNA [NM_001098169]	2,578	5,53E-04
ENST00000405749	A_33_P3351836	ens|Uncharacterized protein C2orf73 [Source:UniProtKB/Swiss-Prot;Acc:Q8N5S3] [ENST00000405749]	2,578	6,18E-04
TAF10	A_33_P3257232	ref|Homo sapiens TAF10 RNA polymerase II, TATA box binding protein (TBP)-associated factor, 30kDa (TAF10), mRNA [NM_006284]	2,575	8,18E-04
ZC3H10	A_24_P318134	ref|Homo sapiens zinc finger CCCH-type containing 10 (ZC3H10), mRNA [NM_032786]	2,572	6,10E-04
BC098409	A_33_P3883985	gb|Homo sapiens cDNA clone IMAGE:5285809. [BC098409]	2,563	1,08E-03
PDZD2	A_33_P3283515	gb|Homo sapiens cDNA FLJ46853 fis, clone UTERU3009775, moderately similar to Rattus norvegicus PAPIN (Papin). [AK128686]	2,554	7,11E-04
TOR2A	A_23_P60534	ref|Homo sapiens torsin family 2, member A (TOR2A), transcript variant 2, mRNA [NM_130459]	2,552	9,92E-04
GPR35	A_33_P3375368	ref|Homo sapiens G protein-coupled receptor 35 (GPR35), mRNA [NM_005301]	2,549	6,02E-04
PCIF1	A_23_P210496	ref|Homo sapiens PDX1 C-terminal inhibiting factor 1 (PCIF1), mRNA [NM_022104]	2,546	5,80E-04
WDR87	A_33_P3388357	ref|Homo sapiens WD repeat domain 87 (WDR87), mRNA [NM_031951]	2,540	6,00E-04
SHISA4	A_23_P115573	ref|Homo sapiens shisa homolog 4 (Xenopus laevis) (SHISA4), transcript variant 1, mRNA [NM_198149]	2,540	5,76E-04
PRB3	A_33_P3256334	ref|Homo sapiens proline-rich protein BstNI subfamily 3 (PRB3), mRNA [NM_006249]	2,539	6,50E-04

FOXP3	A_33_P3398251	ref|Homo sapiens forkhead box P3 (FOXP3), transcript variant 1, mRNA [NM_014009]	2,537	5,67E-04
A_33_P3371144	A_33_P3371144	Unknown	2,535	5,54E-04
LOC100130954	A_33_P3267731	gb|full-length cDNA clone CS0DE013YN04 of Placenta of Homo sapiens (human). [CR619051]	2,529	7,12E-04
MUC4	A_33_P3417281	ref|Homo sapiens mucin 4, cell surface associated (MUC4), transcript variant 1, mRNA [NM_018406]	2,527	7,79E-04
LOC389033	A_33_P3283601	ref|Homo sapiens placenta-specific 9 pseudogene (LOC389033), non-coding RNA [NR_026740]	2,515	5,89E-04
C8orf58	A_33_P3410849	ref|Homo sapiens chromosome 8 open reading frame 58 (C8orf58), mRNA [NM_001013842]	2,503	7,39E-04
SNAR-C3	A_33_P3872301	gb|602075775F1 NIH_MGC_62 Homo sapiens cDNA clone IMAGE:4242834 5', mRNA sequence [BF570763]	2,499	6,52E-04
YJEFN3	A_33_P3382944	ref|Homo sapiens YjeF N-terminal domain containing 3 (YJEFN3), nuclear gene encoding mitochondrial protein, mRNA [NM_198537]	2,499	1,12E-03
C16orf89	A_33_P3296862	ref|Homo sapiens chromosome 16 open reading frame 89 (C16orf89), transcript variant 1, mRNA [NM_152459]	2,495	6,77E-04
KRTAP10-12	A_33_P3357651	ref|Homo sapiens keratin associated protein 10-12 (KRTAP10-12), mRNA [NM_198699]	2,487	6,87E-04
FAM27L	A_32_P942508	ref|Homo sapiens family with sequence similarity 27-like (FAM27L), non-coding RNA [NR_028336]	2,480	6,39E-04
ARHGAP33	A_33_P3313411	ref|Homo sapiens Rho GTPase activating protein 33 (ARHGAP33), transcript variant 2, mRNA [NM_001172630]	2,478	6,81E-04
BRF1	A_33_P3363016	ref|Homo sapiens BRF1 homolog, subunit of RNA polymerase III transcription initiation factor IIIB (S. cerevisiae) (BRF1), transcript variant 1, mRNA [NM_001519]	2,473	6,24E-04
LOC727872	A_33_P3384312	ref|PREDICTED: Homo sapiens hypothetical LOC727872 (LOC727872), mRNA [XM_001720692]	2,462	1,00E-03
A_33_P3332627	A_33_P3332627	Unknown	2,461	6,73E-04
DERL3	A_33_P3724155	ref|Homo sapiens Der1-like domain family, member 3 (DERL3), transcript variant 3, mRNA [NM_198440]	2,458	6,66E-04
MUC4	A_24_P239177	ref|Homo sapiens mucin 4, cell surface associated (MUC4), transcript variant 1, mRNA [NM_018406]	2,453	6,05E-04
LOC100132658	A_24_P246777	gb|PREDICTED: Homo sapiens similar to TGF beta-inducible nuclear protein 1 (LOC100132658), miscRNA [XR_038575]	2,451	6,87E-04

RAD23A	A_33_P3362193	ref|Homo sapiens RAD23 homolog A (S. cerevisiae) (RAD23A), mRNA [NM_005053]	2,448	6,31E-04
C21orf88	A_24_P267664	ref|Homo sapiens chromosome 21 open reading frame 88 (C21orf88), transcript variant 2, non-coding RNA [NR_026543]	2,448	7,44E-04
GPR152	A_33_P3243429	ref|Homo sapiens G protein-coupled receptor 152 (GPR152), mRNA [NM_206997]	2,447	5,94E-04
CDX1	A_33_P3289848	ref|Homo sapiens caudal type homeobox 1 (CDX1), mRNA [NM_001804]	2,444	8,04E-04
VSIG10L	A_23_P101246	ref|Homo sapiens V-set and immunoglobulin domain containing 10 like (VSIG10L), mRNA [NM_001163922]	2,444	5,93E-04
NEAT1	A_33_P3263538	gb|Human MEN1 region clone epsilon/beta mRNA, 3' fragment. [AF001893]	2,439	5,90E-04
CRELD2	A_23_P33465	ref|Homo sapiens cysteine-rich with EGF-like domains 2 (CRELD2), transcript variant 2, mRNA [NM_024324]	2,429	7,35E-04
C19orf48	A_33_P3288754	ref|Homo sapiens chromosome 19 open reading frame 48 (C19orf48), mRNA [NM_199249]	2,427	1,15E-03
ARHGEF18	A_33_P3378915	ref|Homo sapiens Rho/Rac guanine nucleotide exchange factor (GEF) 18 (ARHGEF18), transcript variant 2, mRNA [NM_001130955]	2,425	6,36E-04
GPS2	A_33_P3608172	ref|Homo sapiens G protein pathway suppressor 2 (GPS2), mRNA [NM_004489]	2,420	9,05E-04
PTAFR	A_33_P3393537	ref|Homo sapiens platelet-activating factor receptor (PTAFR), transcript variant 2, mRNA [NM_001164722]	2,419	5,69E-04
ACE	A_33_P3229288	ref|Homo sapiens angiotensin I converting enzyme (peptidyl-dipeptidase A) 1 (ACE), transcript variant 1, mRNA [NM_000789]	2,413	9,41E-04
DUOXA1	A_33_P3410235	ref|Homo sapiens dual oxidase maturation factor 1 (DUOXA1), mRNA [NM_144565]	2,413	7,69E-04
IGLON5	A_24_P271149	ref|Homo sapiens IgLON family member 5 (IGLON5), mRNA [NM_001101372]	2,407	9,04E-04
LOC646396	A_33_P3259861	gb|PREDICTED: Homo sapiens similar to hCG2042704 (LOC646396), mRNA [XM_001726976]	2,399	7,54E-04
A_33_P3269380	A_33_P3269380	Unknown	2,398	5,81E-04
A_33_P3409631	A_33_P3409631	Unknown	2,398	6,26E-04
TEAD3	A_23_P82000	ref|Homo sapiens TEA domain family member 3 (TEAD3), mRNA [NM_003214]	2,396	6,41E-04

P2RX1	A_23_P372848	ref|Homo sapiens purinergic receptor P2X, ligand-gated ion channel, 1 (P2RX1), mRNA [NM_002558]	2,394	1,18E-03
MUC4	A_24_P239176	ref|Homo sapiens mucin 4, cell surface associated (MUC4), transcript variant 1, mRNA [NM_018406]	2,394	6,19E-04
ADAMTS7	A_23_P11005	ref|Homo sapiens ADAM metallopeptidase with thrombospondin type 1 motif, 7 (ADAMTS7), mRNA [NM_014272]	2,394	1,73E-03
KIAA1324	A_33_P3256793	ref|Homo sapiens KIAA1324 (KIAA1324), mRNA [NM_020775]	2,390	7,68E-04
LOC729370	A_33_P3369731	ref|PREDICTED: Homo sapiens hypothetical LOC729370 (LOC729370), mRNA [XM_001130069]	2,385	1,08E-03
WDR1	A_23_P213000	ref|Homo sapiens WD repeat domain 1 (WDR1), transcript variant 1, mRNA [NM_017491]	2,383	8,46E-04
CHKA	A_23_P124742	ref|Homo sapiens choline kinase alpha (CHKA), transcript variant 1, mRNA [NM_001277]	2,378	8,21E-04
LOC100131326	A_33_P3360326	gb|AGENCOURT_8882055 Lupski_sciatic_nerve Homo sapiens cDNA clone IMAGE:6197768 5', mRNA sequence [BQ932264]	2,374	7,74E-04
MLL4	A_33_P3221761	ref|Homo sapiens myeloid/lymphoid or mixed-lineage leukemia 4 (MLL4), mRNA [NM_014727]	2,372	7,02E-04
UPK3A	A_24_P348594	ref|Homo sapiens uroplakin 3A (UPK3A), transcript variant 1, mRNA [NM_006953]	2,369	1,32E-03
COL11A2	A_33_P3216442	ref|Homo sapiens collagen, type XI, alpha 2 (COL11A2), transcript variant 1, mRNA [NM_080680]	2,369	7,19E-04
DIABLO	A_33_P3330125	ref|Homo sapiens diablo homolog (Drosophila) (DIABLO), nuclear gene encoding mitochondrial protein, transcript variant 3, mRNA [NM_138929]	2,367	3,90E-03
ENST00000342518	A_33_P3355296	ens|D-2-hydroxyglutarate dehydrogenase [Source:HGNC Symbol;Acc:28358] [ENST00000342518]	2,365	5,56E-04
**LGALS3**	**A_33_P3306163**	**ref|Homo sapiens lectin, galactoside-binding, soluble, 3 (LGALS3), transcript variant 1, mRNA [NM_002306]**	**2,365**	**7,92E-04**
SPRR1A	A_23_P348208	ref|Homo sapiens small proline-rich protein 1A (SPRR1A), mRNA [NM_005987]	2,363	6,23E-04
CCDC64B	A_33_P3335590	ref|Homo sapiens coiled-coil domain containing 64B (CCDC64B), mRNA [NM_001103175]	2,357	5,87E-04
ENST00000331828	A_33_P3401169	ens|T cell receptor beta variable 21/OR9-2 (non-functional) [Source:HGNC Symbol;Acc:12199] [ENST00000331828]	2,356	6,34E-04
A_33_P3262537	A_33_P3262537	Unknown	2,352	5,99E-04

C19orf69	A_33_P3325634	ref|Homo sapiens chromosome 19 open reading frame 69 (C19orf69), mRNA [NM_001130514]	2,351	7,79E-04
GPD1	A_23_P204736	ref|Homo sapiens glycerol-3-phosphate dehydrogenase 1 (soluble) (GPD1), mRNA [NM_005276]	2,347	6,17E-04
LOC440839	A_33_P3383287	ref|Homo sapiens tigger transposable element derived 1 pseudogene (LOC440839), non-coding RNA [NR_029399]	2,346	8,30E-04
KRTAP12-2	A_33_P3354404	ref|Homo sapiens keratin associated protein 12-2 (KRTAP12-2), mRNA [NM_181684]	2,343	6,07E-04
BGN	A_23_P34126	ref|Homo sapiens biglycan (BGN), mRNA [NM_001711]	2,339	5,59E-04
ENST00000411873	A_23_P333129	ens|D4S2463 homeobox-like Fragment [Source:UniProtKB/TrEMBL;Acc:Q13060] [ENST00000411873]	2,336	7,50E-04
A_33_P3306526	A_33_P3306526	Unknown	2,336	1,21E-03
ZSCAN12L1	A_33_P3422113	ref|Homo sapiens zinc finger and SCAN domain containing 12-like 1 (ZSCAN12L1), non-coding RNA [NR_024063]	2,335	6,89E-04
KRT81	A_33_P3273534	ref|Homo sapiens keratin 81 (KRT81), mRNA [NM_002281]	2,331	6,22E-04
SIGLECP3	A_33_P3309621	ref|Homo sapiens sialic acid binding Ig-like lectin, pseudogene 3 (SIGLECP3), non-coding RNA [NR_002804]	2,330	1,91E-03
C17orf91	A_24_P253723	ref|Homo sapiens chromosome 17 open reading frame 91 (C17orf91), transcript variant 1, non-coding RNA [NR_028502]	2,330	1,76E-03
ZSCAN5A	A_23_P90233	ref|Homo sapiens zinc finger and SCAN domain containing 5A (ZSCAN5A), mRNA [NM_024303]	2,324	6,92E-04
SLC25A16	A_33_P3275959	gb|Homo sapiens solute carrier family 25 (mitochondrial carrier; Graves disease autoantigen), member 16, mRNA (cDNA clone IMAGE:3139311), with apparent retained intron. [BC001407]	2,322	5,97E-04
FGFRL1	A_23_P92349	ref|Homo sapiens fibroblast growth factor receptor-like 1 (FGFRL1), transcript variant 1, mRNA [NM_001004356]	2,317	7,61E-04
NCOA4	A_33_P3319463	ref|Homo sapiens nuclear receptor coactivator 4 (NCOA4), transcript variant 2, mRNA [NM_001145261]	2,315	1,16E-03
AK097701	A_33_P3238521	gb|Homo sapiens cDNA FLJ40382 fis, clone TESTI2035775. [AK097701]	2,308	7,45E-04
KRTAP9L2	A_33_P3358908	ens|keratin associated protein 9-like 2 [Source:HGNC Symbol;Acc:37140] [ENST00000391355]	2,305	6,55E-04
LOC400558	A_33_P3388588	ens|cDNA FLJ27068 fis, clone SPL01475 [Source:UniProtKB/TrEMBL;Acc:Q6ZNV2] [ENST00000378340]	2,303	6,74E-04

SENP1	A_33_P3304528	ref|Homo sapiens SUMO1/sentrin specific peptidase 1 (SENP1), mRNA [NM_014554]	2,302	5,85E-04
LOC645435	A_33_P3362952	gb|Homo sapiens cDNA FLJ43993 fis, clone TESTI4020102. [AK125981]	2,302	5,76E-04
SORBS3	A_33_P3409625	ref|Homo sapiens sorbin and SH3 domain containing 3 (SORBS3), transcript variant 1, mRNA [NM_005775]	2,300	6,09E-04
LOC100133075	A_32_P79115	gb|PREDICTED: Homo sapiens hypothetical LOC100133075 (LOC100133075), miscRNA [XR_039086]	2,296	5,88E-04
ENST00000482292	A_33_P3264444	ens|Prefoldin subunit 6 (Protein Ke2) [Source:UniProtKB/Swiss-Prot;Acc:O15212] [ENST00000482292]	2,293	7,03E-04
PLVAP	A_23_P56328	ref|Homo sapiens plasmalemma vesicle associated protein (PLVAP), mRNA [NM_031310]	2,290	1,05E-03
HDAC4	A_23_P210048	ref|Homo sapiens histone deacetylase 4 (HDAC4), mRNA [NM_006037]	2,288	9,65E-04
ENST00000399801	A_33_P3275826	ens|Putative uncharacterized protein ENSP00000382699 Fragment [Source:UniProtKB/TrEMBL;Acc:A8MYP0] [ENST00000399801]	2,286	1,43E-03
ATM	A_33_P3218410	gb|Homo sapiens ataxia telangiectasia mutated (includes complementation groups A, C and D), mRNA (cDNA clone IMAGE:3950747). [BC007023]	2,283	7,76E-04
MAFIP	A_33_P3406828	gb|Homo sapiens cDNA FLJ39633 fis, clone SMINT2002457, weakly similar to Tektin A1. [AK096952]	2,279	2,00E-03
A_33_P3237605	A_33_P3237605	Unknown	2,272	5,98E-04
KRTAP10-9	A_33_P3293753	ref|Homo sapiens keratin associated protein 10-9 (KRTAP10-9), mRNA [NM_198690]	2,268	5,71E-04
FAM120C	A_33_P3303865	ref|Homo sapiens family with sequence similarity 120C (FAM120C), mRNA [NM_017848]	2,260	6,45E-04
ABCA4	A_23_P160940	ref|Homo sapiens ATP-binding cassette, sub-family A (ABC1), member 4 (ABCA4), mRNA [NM_000350]	2,259	6,56E-04
MAP3K6	A_33_P3404418	ref|Homo sapiens mitogen-activated protein kinase kinase kinase 6 (MAP3K6), mRNA [NM_004672]	2,258	6,81E-04
FLJ33630	A_33_P3407445	gb|full-length cDNA clone CS0DG006YI17 of B cells (Ramos cell line) of Homo sapiens (human). [CR596183]	2,253	8,23E-04
THRA	A_24_P262407	ref|Homo sapiens thyroid hormone receptor, alpha (erythroblastic leukemia viral (v-erb-a) oncogene homolog, avian) (THRA), transcript variant 1, mRNA [NM_199334]	2,252	6,96E-04
C1orf183	A_24_P336577	ref|Homo sapiens chromosome 1 open reading frame 183 (C1orf183), transcript variant 1, mRNA [NM_019099]	2,248	1,27E-03

LOC100128107	A_33_P3272347	gb|Homo sapiens cDNA FLJ41726 fis, clone HLUNG2014449. [AK123720]	2,230	5,83E-04
ENTPD2	A_33_P3278649	ref|Homo sapiens ectonucleoside triphosphate diphosphohydrolase 2 (ENTPD2), transcript variant 1, mRNA [NM_203468]	2,228	6,42E-04
ATP13A2	A_33_P3393806	gb|Homo sapiens unknown mRNA. [AY987009]	2,225	7,53E-04
SNORA10	A_33_P3563369	gb|CM1-HT1146-090101-713-g10 HT1146 Homo sapiens cDNA, mRNA sequence [BG987537]	2,220	9,99E-04
FAM180B	A_33_P3330404	ref|Homo sapiens family with sequence similarity 180, member B (FAM180B), mRNA [NM_001164379]	2,218	6,25E-04
NCRNA00176	A_33_P3403082	ref|Homo sapiens non-protein coding RNA 176 (NCRNA00176), transcript variant 1, non-coding RNA [NR_027686]	2,216	8,02E-04
ADAMTSL5	A_24_P22939	ref|Homo sapiens ADAMTS-like 5 (ADAMTSL5), mRNA [NM_213604]	2,214	9,69E-04
CCDC50	A_24_P910733	ref|Homo sapiens coiled-coil domain containing 50 (CCDC50), transcript variant 2, mRNA [NM_178335]	2,208	5,84E-04
SLC39A5	A_33_P3385006	ref|Homo sapiens solute carrier family 39 (metal ion transporter), member 5 (SLC39A5), transcript variant 2, mRNA [NM_001135195]	2,208	1,00E-03
MT3	A_23_P129629	ref|Homo sapiens metallothionein 3 (MT3), mRNA [NM_005954]	2,203	8,31E-04
**PTP4A3**	**A_33_P3315906**	**ref|Homo sapiens protein tyrosine phosphatase type IVA, member 3 (PTP4A3), transcript variant 1, mRNA [NM_032611]**	**2,201**	**1,16E-03**
CHRNA4	A_33_P3217584	ref|Homo sapiens cholinergic receptor, nicotinic, alpha 4 (CHRNA4), mRNA [NM_000744]	2,199	9,30E-04
SNAR-C4	A_33_P3542911	gb|602076150F1 NIH_MGC_62 Homo sapiens cDNA clone IMAGE:4243164 5', mRNA sequence [BF570948]	2,197	5,95E-04
FAM27A	A_33_P3412311	gb|Homo sapiens cDNA FLJ36039 fis, clone TESTI2017311. [AK093358]	2,195	6,65E-04
AK299501	A_33_P3219010	gb|Homo sapiens cDNA FLJ61160 complete cds. [AK299501]	2,192	1,31E-03
CREB3L3	A_23_P108082	ref|Homo sapiens cAMP responsive element binding protein 3-like 3 (CREB3L3), mRNA [NM_032607]	2,191	7,63E-04
CCL19	A_23_P123853	ref|Homo sapiens chemokine (C-C motif) ligand 19 (CCL19), mRNA [NM_006274]	2,176	7,25E-04
PGM5	A_24_P254949	ref|Homo sapiens phosphoglucomutase 5 (PGM5), mRNA [NM_021965]	2,175	7,32E-04

ENST00000383146	A_33_P3391345	ens|Putative uncharacterized protein ENSP00000384224 [Source:UniProtKB/TrEMBL;Acc:B5MBW5] [ENST00000383146]	2,175	7,64E-04
LOC285957	A_33_P3734384	gb|Homo sapiens cDNA FLJ40207 fis, clone TESTI2020946. [AK097526]	2,171	7,56E-04
ENST00000447029	A_33_P3628409	ens|Putative uncharacterized protein DKFZp434H1419 Fragment [Source:UniProtKB/TrEMBL;Acc:Q9NT46] [ENST00000447029]	2,167	1,39E-03
MTCH1	A_33_P3379157	ref|Homo sapiens mitochondrial carrier homolog 1 (C. elegans) (MTCH1), nuclear gene encoding mitochondrial protein, mRNA [NM_014341]	2,163	1,01E-03
QSOX1	A_33_P3225268	ref|Homo sapiens quiescin Q6 sulfhydryl oxidase 1 (QSOX1), transcript variant 2, mRNA [NM_001004128]	2,163	2,18E-03
KRT8	A_33_P3372368	gb|Homo sapiens cDNA, FLJ79475 complete cds, highly similar to Keratin, type II cytoskeletal 8. [AK315826]	2,159	6,69E-04
A_33_P3350549	A_33_P3350549	Unknown	2,158	8,71E-04
LOC100128348	A_33_P3283061	gb|Homo sapiens cDNA FLJ46249 fis, clone TESTI4021377. [AK128128]	2,155	7,13E-04
SCXA	A_33_P3299510	ref|Homo sapiens scleraxis homolog A (mouse) (SCXA), mRNA [NM_001008271]	2,152	8,44E-04
ARID5A	A_33_P3389342	ref|Homo sapiens AT rich interactive domain 5A (MRF1-like) (ARID5A), mRNA [NM_212481]	2,150	9,83E-04
LOC100130453	A_33_P3401841	gb|Homo sapiens cDNA FLJ41926 fis, clone PERIC2003834. [AK123920]	2,144	1,26E-03
TBC1D10B	A_23_P22382	ref|Homo sapiens TBC1 domain family, member 10B (TBC1D10B), mRNA [NM_015527]	2,137	9,06E-04
LOC151009	A_33_P3228609	ref|Homo sapiens hypothetical LOC151009 (LOC151009), non-coding RNA [NR_027244]	2,133	1,34E-03
CASC2	A_32_P170621	ref|Homo sapiens cancer susceptibility candidate 2 (CASC2), transcript variant 3, non-coding RNA [NR_026941]	2,131	9,21E-04
CCDC78	A_33_P3246613	ref|Homo sapiens coiled-coil domain containing 78 (CCDC78), mRNA [NM_001031737]	2,130	1,79E-03
PRO0628	A_33_P3324137	ref|Homo sapiens hypothetical LOC29053 (PRO0628), non-coding RNA [NR_002764]	2,130	5,85E-04
CTRB2	A_32_P86150	ref|Homo sapiens chymotrypsinogen B2 (CTRB2), mRNA [NM_001025200]	2,129	1,01E-03
SLC11A1	A_33_P3702055	ref|Homo sapiens solute carrier family 11 (proton-coupled divalent metal ion transporters), member 1 (SLC11A1), mRNA [NM_000578]	2,125	1,05E-03

GJD4	A_33_P3353496	ref|Homo sapiens gap junction protein, delta 4, 40.1kDa (GJD4), mRNA [NM_153368]	2,124	2,33E-03
AX747383	A_33_P3868357	gb|Sequence 908 from Patent EP1308459. [AX747383]	2,114	6,68E-04
KANK3	A_33_P3326025	gb|Homo sapiens LP2709 mRNA, complete cds. [AY203940]	2,112	6,32E-04
ANKMY1	A_33_P3629247	ref|Homo sapiens ankyrin repeat and MYND domain containing 1 (ANKMY1), transcript variant 2, mRNA [NM_017844]	2,111	1,76E-03
FAM99A	A_33_P3378785	gb|Homo sapiens cDNA FLJ42833 fis, clone BRCAN2018935. [AK124823]	2,111	2,01E-03
CU678501	A_32_P93036	gb|Synthetic construct Homo sapiens gateway clone IMAGE:100023427 3' read APOE mRNA. [CU678501]	2,110	8,34E-04
ENST00000413098	A_33_P3236748	ens|Putative uncharacterized protein UNQ9370/PRO34162 [Source:UniProtKB/Swiss-Prot;Acc:Q6UXP9] [ENST00000413098]	2,109	7,61E-04
ENST00000397526	A_33_P3379056	ens|Putative uncharacterized protein ENSP00000380660 Fragment [Source:UniProtKB/TrEMBL;Acc:A8MYS4] [ENST00000397526]	2,106	7,36E-04
ENST00000390291	A_24_P318990	ens|immunoglobulin lambda variable 1-50 (non-functional) [Source:HGNC Symbol;Acc:5881] [ENST00000390291]	2,101	7,72E-04
GAMT	A_24_P19228	ref|Homo sapiens guanidinoacetate N-methyltransferase (GAMT), transcript variant 2, mRNA [NM_138924]	2,100	9,55E-04
CASS4	A_33_P3228931	ref|Homo sapiens Cas scaffolding protein family member 4 (CASS4), transcript variant 4, mRNA [NM_001164115]	2,098	7,59E-04
LOC645321	A_33_P3347663	gb|PREDICTED: Homo sapiens hypothetical LOC645321 (LOC645321), mRNA [XM_001714545]	2,097	1,02E-03
MARK4	A_33_P3322415	ref|Homo sapiens MAP/microtubule affinity-regulating kinase 4 (MARK4), mRNA [NM_031417]	2,096	5,82E-04
HCN2	A_33_P3279640	ref|Homo sapiens hyperpolarization activated cyclic nucleotide-gated potassium channel 2 (HCN2), mRNA [NM_001194]	2,093	1,71E-03
DLK2	A_33_P3254460	ref|Homo sapiens delta-like 2 homolog (Drosophila) (DLK2), transcript variant 2, mRNA [NM_206539]	2,088	6,14E-04
ENST00000370222	A_33_P3360758	ens|potassium voltage-gated channel, KQT-like subfamily, member 2 [Source:HGNC Symbol;Acc:6296] [ENST00000370222]	2,082	6,05E-04
ZMYND15	A_23_P89570	ref|Homo sapiens zinc finger, MYND-type containing 15 (ZMYND15), transcript variant 2, mRNA [NM_032265]	2,082	9,82E-04
CORO6	A_23_P107322	ref|Homo sapiens coronin 6 (CORO6), mRNA [NM_032854]	2,080	6,82E-04

LOC728846	A_33_P3417176	gb|PREDICTED: Homo sapiens hypothetical LOC728846 (LOC728846), mRNA [XM_001715013]	2,078	3,37E-03
CLTCL1	A_23_P143559	ref|Homo sapiens clathrin, heavy chain-like 1 (CLTCL1), transcript variant 1, mRNA [NM_007098]	2,072	7,23E-04
HMX1	A_33_P3279009	ref|Homo sapiens H6 family homeobox 1 (HMX1), mRNA [NM_018942]	2,069	6,59E-04
LOC284232	A_32_P150086	ref|Homo sapiens ankyrin repeat domain 20 family, member A2 pseudogene (LOC284232), non-coding RNA [NR_027995]	2,069	8,93E-04
LOC100132288	A_23_P356646	ref|Homo sapiens hypothetical protein LOC100132288 (LOC100132288), mRNA [NM_001033515]	2,068	8,64E-04
BC171856	A_33_P3290748	gb|Homo sapiens cDNA clone IMAGE:9054510. [BC171856]	2,068	5,81E-04
SEPT12	A_23_P89101	ref|Homo sapiens septin 12 (SEPT12), transcript variant 2, mRNA [NM_144605]	2,068	1,37E-03
A_33_P3313940	A_33_P3313940	Unknown	2,063	6,56E-04
LRP3	A_23_P16415	ref|Homo sapiens low density lipoprotein receptor-related protein 3 (LRP3), mRNA [NM_002333]	2,060	9,67E-04
A_33_P3396434	A_33_P3396434	Unknown	2,056	1,27E-03
NKX2-5	A_33_P3299599	ref|Homo sapiens NK2 transcription factor related, locus 5 (Drosophila) (NKX2-5), transcript variant 2, mRNA [NM_001166175]	2,050	1,84E-03
LOC284440	A_33_P3251640	ref|Homo sapiens hypothetical LOC284440 (LOC284440), non-coding RNA [NR_026956]	2,050	1,24E-03
A_33_P3287388	A_33_P3287388	Unknown	2,048	1,08E-03
LOC100132790	A_33_P3265593	gb|Homo sapiens cDNA FLJ44869 fis, clone BRAMY2015516. [AK128756]	2,048	6,60E-04
FAM63B	A_23_P379327	ref|Homo sapiens family with sequence similarity 63, member B (FAM63B), transcript variant 1, mRNA [NM_001040450]	2,047	1,22E-03
LPHN1	A_23_P391926	ref|Homo sapiens latrophilin 1 (LPHN1), transcript variant 1, mRNA [NM_001008701]	2,042	1,72E-03
SGK3	A_33_P3375613	ref|Homo sapiens serum/glucocorticoid regulated kinase family, member 3 (SGK3), transcript variant 1, mRNA [NM_013257]	2,033	1,78E-03
ALKBH2	A_32_P28939	ref|Homo sapiens alkB, alkylation repair homolog 2 (E. coli) (ALKBH2), transcript variant 2, mRNA [NM_001001655]	2,031	1,19E-03

A_33_P3293645	A_33_P3293645	Unknown	2,026	8,29E-04
LOC100133402	A_33_P3385351	ref|PREDICTED: Homo sapiens hypothetical LOC100133402 (LOC100133402), mRNA [XM_001718349]	2,025	1,70E-03
C4orf23	A_23_P29985	gb|Homo sapiens cDNA FLJ12891 fis, clone NT2RP2004142. [AK022953]	2,011	7,29E-04
LOC440330	A_32_P351277	gb|Homo sapiens, clone IMAGE:3543963, mRNA. [BC004968]	2,001	1,58E-03
ENO3	A_23_P130149	ref|Homo sapiens enolase 3 (beta, muscle) (ENO3), transcript variant 1, mRNA [NM_001976]	2,000	7,86E-04
APOL1	A_24_P87931	ref|Homo sapiens apolipoprotein L, 1 (APOL1), transcript variant 2, mRNA [NM_145343]	1,992	2,04E-03
CTSC	A_23_P1552	ref|Homo sapiens cathepsin C (CTSC), transcript variant 1, mRNA [NM_001814]	1,992	1,04E-03
A_33_P3318766	A_33_P3318766	Unknown	1,982	9,11E-04
ZNF527	A_33_P3328154	gb|Homo sapiens zinc finger protein 527, mRNA (cDNA clone IMAGE:3931715), complete cds. [BC014325]	1,978	1,89E-03
MDFI	A_33_P3246418	ref|Homo sapiens MyoD family inhibitor (MDFI), mRNA [NM_005586]	1,970	2,70E-03
GALR3	A_23_P256663	ref|Homo sapiens galanin receptor 3 (GALR3), mRNA [NM_003614]	1,968	1,71E-03
LOC645553	A_33_P3454968	gb|Homo sapiens cDNA clone IMAGE:5478640, partial cds. [BC062328]	1,968	7,48E-04
GP9	A_33_P3383226	ref|Homo sapiens glycoprotein IX (platelet) (GP9), mRNA [NM_000174]	1,948	1,17E-03
LOC147804	A_33_P3216433	ref|Homo sapiens tropomyosin 3 pseudogene (LOC147804), non-coding RNA [NR_003148]	1,942	1,22E-03
AK094832	A_33_P3279620	gb|Homo sapiens cDNA FLJ37513 fis, clone BRCAN2000620. [AK094832]	1,942	1,44E-03
LOC100127955	A_33_P3399693	gb|Homo sapiens cDNA FLJ45105 fis, clone BRAWH3033117. [AK127048]	1,935	9,89E-04
DB066855	A_33_P3308774	gb|DB066855 TESTI4 Homo sapiens cDNA clone TESTI4008311 5', mRNA sequence [DB066855]	1,934	7,95E-04
SPSB4	A_23_P258381	ref|Homo sapiens splA/ryanodine receptor domain and SOCS box containing 4 (SPSB4), mRNA [NM_080862]	1,923	6,88E-04

KLC2	A_33_P3219895	ref|Homo sapiens kinesin light chain 2 (KLC2), transcript variant 2, mRNA [NM_001134774]	1,917	8,87E-04
SFTA1P	A_33_P3474175	gb|AGENCOURT_10018270 NIH_MGC_142 Homo sapiens cDNA clone IMAGE:6495000 5', mRNA sequence [BU601128]	1,914	1,79E-03
IQSEC2	A_32_P159445	ref|Homo sapiens IQ motif and Sec7 domain 2 (IQSEC2), transcript variant 2, mRNA [NM_015075]	1,907	1,56E-03
RPL5	A_23_P12140	ref|Homo sapiens ribosomal protein L5 (RPL5), mRNA [NM_000969]	1,906	2,81E-03
A_33_P3303557	A_33_P3303557	Unknown	1,898	6,97E-04
A_33_P3309849	A_33_P3309849	Unknown	1,896	1,15E-03
APBB2	A_33_P3251901	ref|Homo sapiens amyloid beta (A4) precursor protein-binding, family B, member 2 (APBB2), transcript variant 1, mRNA [NM_004307]	1,882	9,24E-04
THC2536940	A_33_P3303430	thc|Q8IWY7_HUMAN (Q8IWY7) Tau-tubulin kinase, partial (6%) [THC2536940]	1,862	6,68E-04
LOC100130600	A_33_P3263603	gb|PREDICTED: Homo sapiens similar to hCG2014367 (LOC100130600), mRNA [XM_001726460]	1,838	2,48E-03
AL050203	A_33_P3291459	gb|Homo sapiens mRNA; cDNA DKFZp586F1123 (from clone DKFZp586F1123). [AL050203]	1,837	6,38E-04
TRIM39	A_33_P3306217	ref|Homo sapiens tripartite motif-containing 39 (TRIM39), transcript variant 2, mRNA [NM_172016]	1,777	1,66E-03
ENST00000281753	A_33_P3410251	ens|unc-80 homolog (C. elegans) [Source:HGNC Symbol;Acc:26582] [ENST00000281753]	1,764	1,45E-03
FNDC4	A_23_P16834	ref|Homo sapiens fibronectin type III domain containing 4 (FNDC4), mRNA [NM_022823]	1,724	1,48E-03
LOC283911	A_33_P3808371	gb|Homo sapiens mRNA; cDNA DKFZp686C2429 (from clone DKFZp686C2429). [AL833480]	1,714	1,36E-03
LOC100129869	A_33_P3236322	gb|Homo sapiens cDNA FLJ46124 fis, clone TESTI2040372. [AK128005]	1,712	3,91E-03
SLC26A1	A_23_P80954	ref|Homo sapiens solute carrier family 26 (sulfate transporter), member 1 (SLC26A1), transcript variant 1, mRNA [NM_022042]	1,704	3,24E-03
AK097351	A_33_P3252181	gb|Homo sapiens cDNA FLJ40032 fis, clone STOMA2009256. [AK097351]	1,626	1,90E-03
A_33_P3216267	A_33_P3216267	Unknown	1,587	2,50E-03

DKFZP547L112	A_33_P3349252	gb|Homo sapiens mRNA; cDNA DKFZp547L112 (from clone DKFZp547L112). [AL512723]	1,583	3,29E-03
IKZF2	A_33_P3328485	ref|Homo sapiens IKAROS family zinc finger 2 (Helios) (IKZF2), transcript variant 2, mRNA [NM_001079526]	1,558	1,67E-03
LOC200830	A_33_P3466016	gb|Homo sapiens cDNA FLJ30391 fis, clone BRACE2008336. [AK054953]	1,545	1,57E-03
AUTS2	A_33_P3330211	ref|Homo sapiens autism susceptibility candidate 2 (AUTS2), transcript variant 3, mRNA [NM_001127232]	1,529	3,71E-03

SUPPLEMENTARY TABLE S4

Probes uniquely detected in NSCLC patients

SNORA71A	A_33_P3495962	gb|AGENCOURT_6611325 NIH_MGC_106 Homo sapiens cDNA clone IMAGE:5485440 5', mRNA sequence [BM918074]
ENST00000400189	A_33_P3372659	ens|Putative uncharacterized protein ENSP00000383050 Fragment [Source:UniProtKB/TrEMBL;Acc:A8MTI4] [ENST00000400189]
NGFRAP1	A_23_P45524	ref|Homo sapiens nerve growth factor receptor (TNFRSF16) associated protein 1 (NGFRAP1), transcript variant 3, mRNA [NM_014380]
C6orf25	A_23_P81934	ref|Homo sapiens chromosome 6 open reading frame 25 (C6orf25), transcript variant 1, mRNA [NM_025260]
**ITGB3**	**A_24_P318656**	**ref|Homo sapiens integrin, beta 3 (platelet glycoprotein IIIa, antigen CD61) (ITGB3), mRNA [NM_000212]**
JAKMIP3	A_24_P332081	ref|Homo sapiens Janus kinase and microtubule interacting protein 3 (JAKMIP3), mRNA [NM_001105521]
KCNMB1	A_24_P206121	ref|Homo sapiens potassium large conductance calcium-activated channel, subfamily M, beta member 1 (KCNMB1), mRNA [NM_004137]
C7orf41	A_32_P100439	ref|Homo sapiens chromosome 7 open reading frame 41 (C7orf41), mRNA [NM_152793]

SUPPLEMENTARY TABLE S5

Univariate Cox regression analysis for CTC markers and clinicopathological parameters

	[redacted]			
**Univariate Cox analysis**	**HR (95% CI)**	**p value**	**HR (95% CI)**	**p value**
GAPDH (poor vs. good prognosis)	5.71 (2.4-13.4)	**<0.001**	9.11 (3.5-23.9)	**<0.001**
NOTCH1 (poor vs. good prognosis)	2.11 (1.0-4.2)	**0.034**	1.16 (0.6-2.4)	0.689
PTP4A3 (poor vs. good prognosis)	3.10 (1.5-6.5)	**0.003**	1.45 (0.7-3.0)	0.305
LGALS3 (poor vs. good prognosis)	2.03 (1.0-4.0)	**0.044**	1.60 (0.8-3.2)	0.193
ITGB3 (poor vs. good prognosis)	2.02 (1.0-4.0)	**0.046**	2.74 (1.3-5.6)	**0.006**
Performance status (PS2 /PS3 vs. PS0/PS1)	1.67 (0.7-3.9)	0.236	1.56 (0.7-3.6)	0.055
Sex (male vs. female)	1.11 (0.5-2.4)	0.792	1.08 (0.4-2.6)	0.863
Histology (squamous vs. adenocarcinoma)	1.45 (0.7-3.0)	0.319	1.08 (0.5-2.4)	0.847
Pathologic status (IIIA/IIIB vs. IV)	1.08 (0.4-2.6)	0.859	1.13 (0.4-2.9)	0.803
T (T3/T4 vs. T0/T1/T2)	1.52 (0.8-3.0)	0.234	1.00 (0.5-2.1)	0.990
N (N3 vs. N0/N1/N2)	1.72 (0.9-3.3)	0.101	1.37 (0.7-2.7)	0.364
No. of metastases (≥2 vs. <2)	1.37 (0.7-2.6)	0.359	1.78 (0.9-3.7)	0.118
Bone metastasis (Y/N)	2.39 (1.2-4.8)	**0.014**	1.82 (0.9-3.7)	0.096
Hepatic metastasis (Y/N)	0.99 (0.4-2.2)	0.980	1.13 (0.5-2.6)	0.777

HR: hazard ratio; CI: confidence interval.

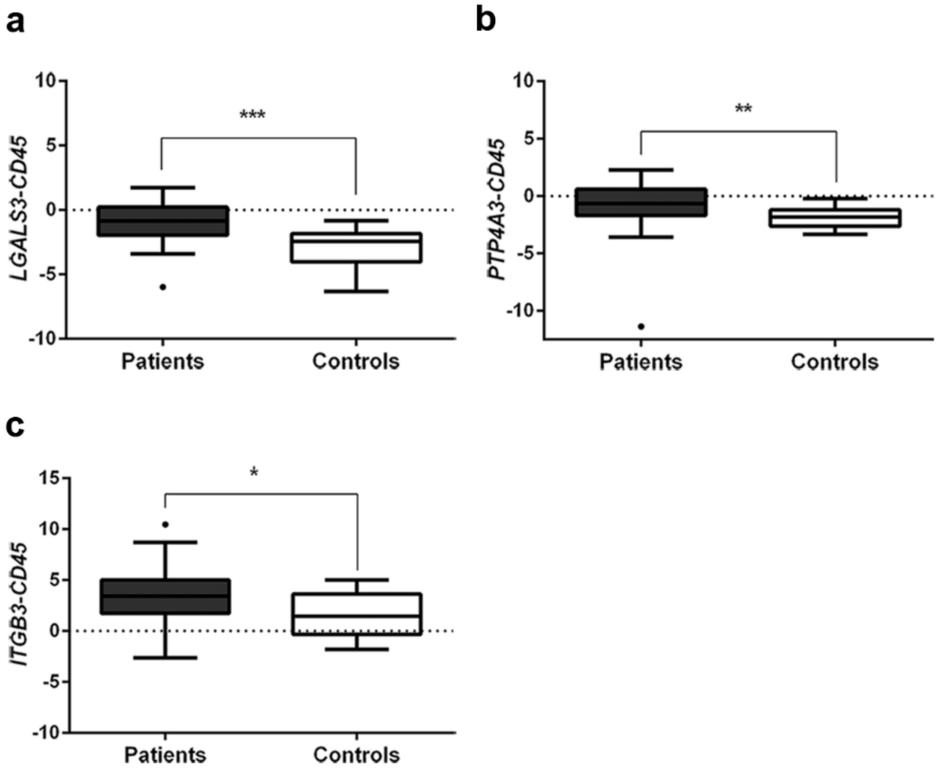
a
b
c
LGALS3-CD45
PTP4A3-CD45
ITGB3-CD45
***
**
*
Patients
Controls

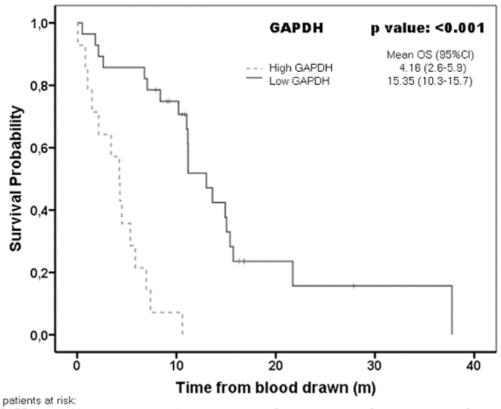

No of patients at risk:

	0	10	20	30	40
High GAPDH:	14	1	0	0	0
Low GAPDH:	28	18	3	1	0

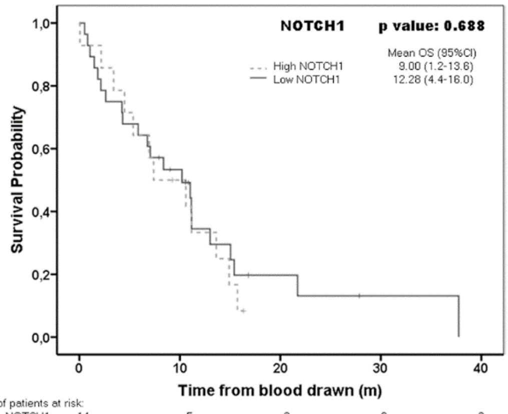

No of patients at risk:

	0	10	20	30	40
High NOTCH1:	14	5	0	0	0
Low NOTCH1:	28	18	3	1	0

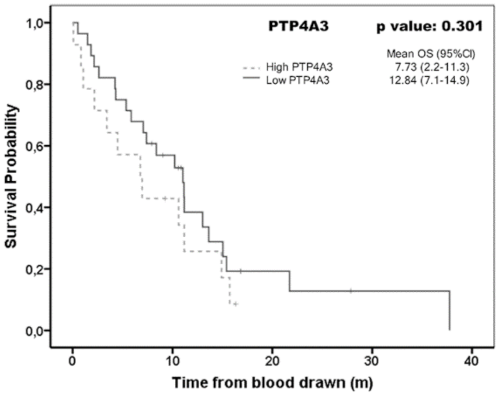

No of patients at risk:

	0	10	20	30	40
High PTP4A3:	14	5	0	0	0
Low PTP4A3:	28	14	3	1	0

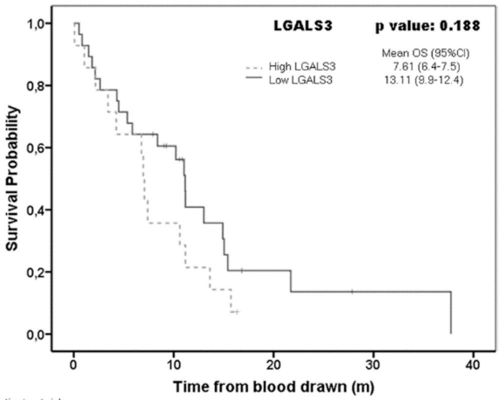

No of patients at risk:

	0	10	20	30	40
High LGALS3:	14	5	0	0	0
Low LGALS3:	28	14	3	1	0

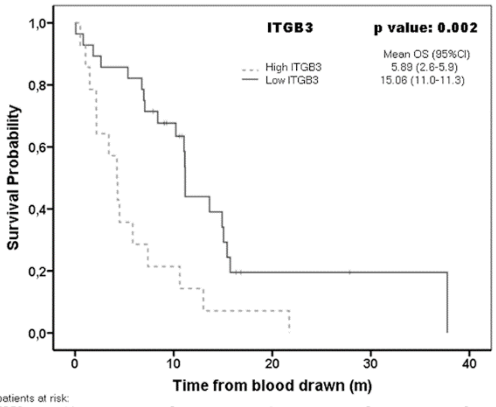

No of patients at risk:

	0	10	20	30	40
High ITGB3:	14	3	1	0	0
Low ITGB3:	28	16	2	1	0

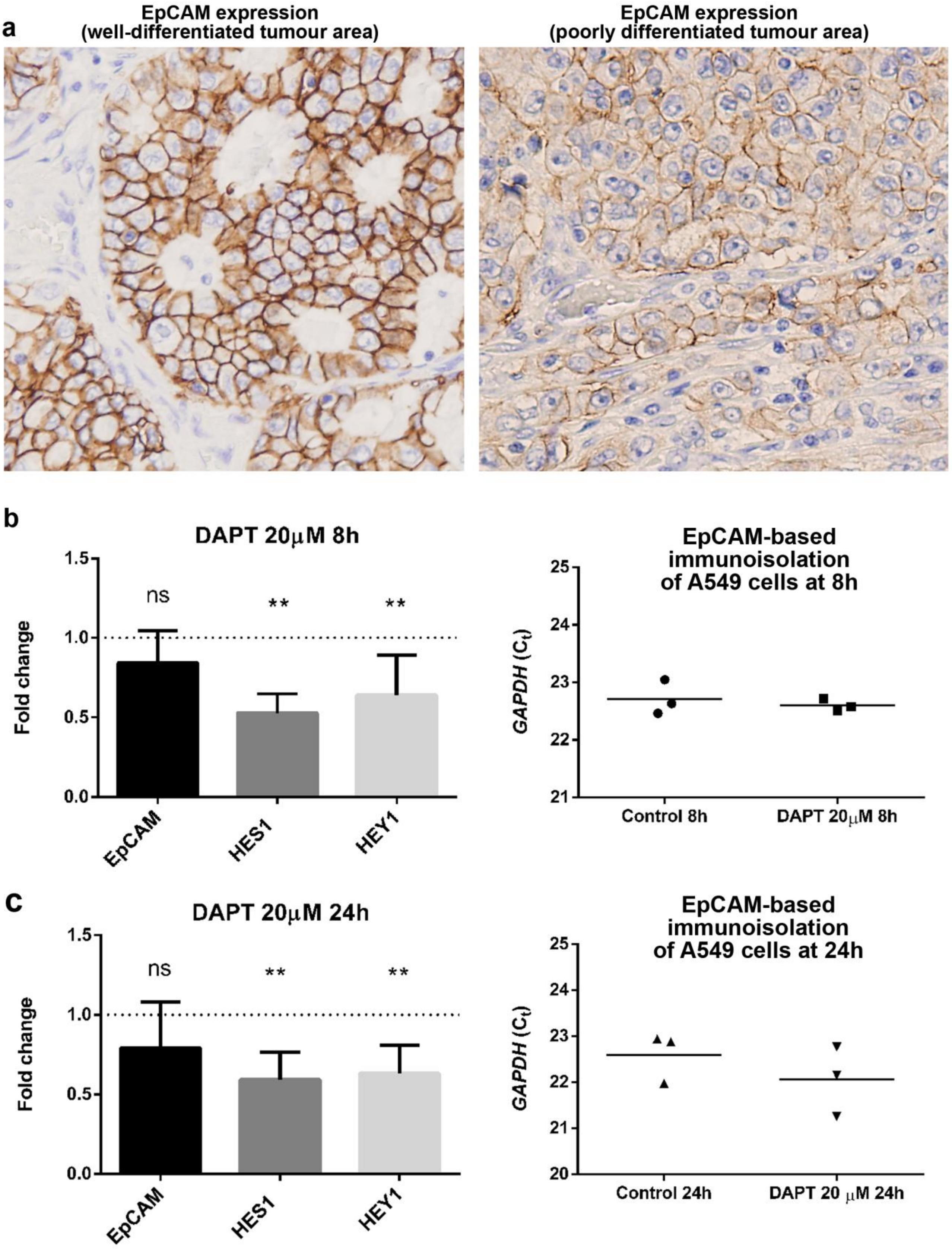
a
EpCAM expression
(well-differentiated tumour area)
EpCAM expression
(poorly differentiated tumour area)
b
DAPT 20μM 8h
Fold change
1.5
1.0
0.5
0.0
ns
**
**
EpCAM
HES1
HEY1
EpCAM-based
immunoisolation
of A549 cells at 8h
GAPDH (Ct)
25
24
23
22
21
Control 8h
DAPT 20μM 8h
c
DAPT 20μM 24h
Fold change
1.5
1.0
0.5
0.0
ns
**
**
EpCAM
HES1
HEY1
EpCAM-based
immunoisolation
of A549 cells at 24h
GAPDH (Ct)
25
24
23
22
21
20
Control 24h
DAPT 20 μM 24h

SUPPLEMENTARY INFORMATION

SUPPLEMENTARY MATERIAL AND METHODS. Extended SAM analysis and TaqMan RT-qPCR probes characteristics. (bp: base pairs).

SUPPLEMENTARY TABLE S1. Clinicopathological characteristics of NSCLC cohort (n=42). The number of metastases was determined by CT scan.

SUPPLEMENTARY TABLE S2. Clinicopathological characteristics of the NSCLC patients used for Agilent gene expression array. Metastases were determined by CT scan.

SUPPLEMENTARY TABLE S3. Gene expression profile of EpCAM-positive CTCs from advanced NSCLC.

SUPPLEMENTARY TABLE S4. Probes uniquely detected in our cohort of patients.

SUPPLEMENTARY TABLE S5. Univariate Cox regression analysis for clinical parameters and CTC markers for PFS and OS (n=42). Poor prognosis group defined based on a 67-33% cut-off criteria.

SUPPLEMENTARY FIGURE S1. Gene expression analysis and validation. Box plot analysis of RT-qPCR expression of candidate genes normalized with *CD45* in CTCs from advanced NSCLC patients and controls. Horizontal bars represent mean and standard deviation. Significant differences can be found between controls (n=16) and patients (n=42) for the candidate genes ($p<0.05$).

SUPPLEMENTARY FIGURE S2. Kaplan-Meier curves of validated biomarkers for Overall Survival (OS). Statistical significance determined by log rank test ($p<0.05$).

SUPPLEMENTARY FIGURE S3. EpCAM expression and EpCAM-based A549 isolation upon Notch inhibition. (a) Immunohistochemical evaluation of EpCAM expression in well (left panel) and poorly differentiated (right panel) areas from a lung adenocarcinoma. **(b and c)** RTqPCR quantification of EpCAM expression (left panels) and A549 lung cancer cells recovery (right panels) after (b) 8 and (c)

24 hours of treatment with Notch inhibitor DAPT (n=3). A549 recovery was determined by *GAPDH* signal by RT-qPCR; HES1 and HEY1 expression in left panels are shown as downstream effectors of Notch pathway, significantly reduced upon DAPT treatment ($p=0.004$; $n=9$). No significant changes in EpCAM expression (left panels) or reduced EpCAM-based immunoisolation (right panels) were observed upon Notch signaling inhibition.
